# Much ado about nothing? Off-target amplification can lead to false-positive bacterial brain microbiome detection in healthy and Parkinson’s disease individuals

**DOI:** 10.1186/s40168-021-01012-1

**Published:** 2021-03-26

**Authors:** Janis R. Bedarf, Naiara Beraza, Hassan Khazneh, Ezgi Özkurt, David Baker, Valeri Borger, Ullrich Wüllner, Falk Hildebrand

**Affiliations:** 1 Department of Neurology, University Hospital Bonn, Venusberg Campus 1, 53127 Bonn, Germany; 2 Gut Microbes & Health, Quadram Institute Bioscience, Norwich Research Park, Norwich, Norfolk, NR4 7UA UK; 3 Earlham Institute, Norwich Research Park, Norwich, Norfolk, NR4 7UA UK; 4 Department of Neurosurgery, University Hospital Bonn, Venusberg Campus 1, 53127 Bonn, Germany; 5 Deutsches Zentrum für Neurodegenerative Erkrankungen (DZNE), Venusberg Campus 2, 53127 Bonn, Germany

**Keywords:** Microbiome, Brain, Brain-microbiome, 16S rRNA gene, Next generation sequencing, Bacterial infection, qPCR

## Abstract

**Background:**

Recent studies suggested the existence of (poly-)microbial infections in human brains. These have been described either as putative pathogens linked to the neuro-inflammatory changes seen in Parkinson’s disease (PD) and Alzheimer’s disease (AD) or as a “brain microbiome” in the context of healthy patients’ brain samples.

**Methods:**

Using 16S rRNA gene sequencing, we tested the hypothesis that there is a bacterial brain microbiome. We evaluated brain samples from healthy human subjects and individuals suffering from PD (olfactory bulb and pre-frontal cortex), as well as murine brains. In line with state-of-the-art recommendations, we included several negative and positive controls in our analysis and estimated total bacterial biomass by 16S rRNA gene qPCR.

**Results:**

Amplicon sequencing did detect bacterial signals in both human and murine samples, but estimated bacterial biomass was extremely low in all samples. Stringent reanalyses implied bacterial signals being explained by a combination of exogenous DNA contamination (54.8%) and false positive amplification of host DNA (34.2%, off-target amplicons). Several seemingly brain-enriched microbes in our dataset turned out to be false-positive signals upon closer examination.

We identified off-target amplification as a major confounding factor in low-bacterial/high-host-DNA scenarios. These amplified human or mouse DNA sequences were clustered and falsely assigned to bacterial taxa in the majority of tested amplicon sequencing pipelines. Off-target amplicons seemed to be related to the tissue’s sterility and could also be found in independent brain 16S rRNA gene sequences.

**Conclusions:**

Taxonomic signals obtained from (extremely) low biomass samples by 16S rRNA gene sequencing must be scrutinized closely to exclude the possibility of off-target amplifications, amplicons that can only appear enriched in biological samples, but are sometimes assigned to bacterial taxa. Sequences must be explicitly matched against any possible background genomes present in large quantities (i.e., the host genome). Using close scrutiny in our approach, we find no evidence supporting the hypothetical presence of either a brain microbiome or a bacterial infection in PD brains.

**Video abstract**

**Supplementary Information:**

The online version contains supplementary material available at 10.1186/s40168-021-01012-1.

## Background

There is growing evidence for a role of gut microbiota and metabolism in many neurodegenerative diseases, such as Parkinson’s disease (PD), amyotrophic lateral sclerosis (ALS), and Alzheimer’s disease (AD) [1–3]. Communication between the gut and the central nervous system (CNS) can be immunological, hematological, or neuronal (*gut-brain-axis*). An even more direct communication route has also been suggested; several studies report the presence of bacterial and/or fungal DNA in post-mortem brains of patients with PD [4], AD [5–8], ALS [9], and multiple sclerosis [10]. Moreover, microscopy studies suggested that bacteria might inhabit healthy human brain astrocytes [11]. While structural signs of CNS inflammation were lacking, previous findings suggest the unappreciated potential for coexistence of bacteria alongside brain cells, i.e., a possible “brain-microbiome”.

Until now, the brain has been viewed as a sterile organ. However, microbiome research in the past decade has considerably increased our knowledge of human organ-specific microbiomes. The lungs and stomach are colonized by specific microbiota, and among others, blood [12] and liver [13] microbiomes have been reported. Currently, there is debate as to whether these are viable, self-sustained communities, or whether they represent microbial “leakage” from other organs. With regard to the gut, dysbiosis in combination with impaired intestinal barrier function could facilitate bacterial translocation from the gut to the enteric nerves or the bloodstream. Thus, hypothetically, bacterial transmission into the CNS via the blood stream, or retrograde axonal transport, could contribute to the immune responses observed in neurodegenerative disorders such as PD (or AD). Alternatively, prior to reaching other brain regions, microbes could enter the CNS via the olfactory bulb, where initiation of the neurodegenerative process is thought to begin, too [14, 15].

Here, we used 16S rRNA gene sequencing to test whether there are bacteria present in the mammalian brain that could be considered either as a *brain microbiome* in healthy individuals or as a brain infection in patients with PD. Considering the hypothesis of a spreading pathology in PD, we investigated samples from different brain regions, commonly affected by the disease pathology obtained from either healthy or PD individuals. We also evaluated specific pathogen-free and germ-free murine brains as an interspecies control.

To ensure a proper interpretation of the data in this “proof of concept” study, our methodology addressed a number of challenges: the expected low bacterial biomass, exogenous DNA contamination, and the large quantity of host DNA present. To overcome these challenges, we implemented stringent pre- and post-sequencing techniques [16], included additional complementary DNA quantification (16S rRNA gene qPCR), positive and negative sequencing controls, and computational procedures to remove contamination.

## Methods

### Human and murine brain tissue

For this proof of concept study, we evaluated *N* = 47 frozen human post-mortem brain samples from PD patients (*n* = 25) and healthy donors (HC, *n* = 22) (ethical vote 074/19). Samples were obtained either from the Netherlands Brain Bank (The Netherlands Brain Bank, Meibergdreef 47, 1105 BA Amsterdam, NBB, *n* = 20 inferior frontal gyrus and *n* = 19 olfactory bulb) or the Munich Brain Bank (Center for Neuropathology and Prion Research, Feodor-Lynen-Str. 23, D-81377 Munich, Germany, *n* = 8 putamen). PD diagnosis had been confirmed using standardized neuropathological methods. Retrospective analysis of metadata (age and post-mortem delay) was performed based on data provided by the respective brain bank. To account for the likely “non-sterile” harvesting of post-mortem brain tissue during autopsy, we also included samples of sterile fresh brain samples (*n* = 3; cortex) from living volunteers (sterile cortex, SC) that were obtained during resective brain surgery (in cooperation with the Dept. of Neurosurgery (VB), University of Bonn; ethical vote 404/17).

We also investigated *n*=5 brain samples that originated from specific pathogen-free wild-type mice (C57Bl6 BDL, 7d, male, SPF), as well as *n*=3 mice held under germ-free conditions (C57Bl6 Germ free, 5 months, male, GRF).

### Pre-sequencing approaches to assess and reduce contamination

To assess and reduce exogenous DNA contamination during tissue handling and experimental procedures, current pre-sequencing recommendations were followed, specifically the “RIDE-criteria” [16] (Table 1). Tissue handling: sterile gloves and lab. coats were used throughout sample handling; wearing sterile gloves, tissues were cut using sterile scalpels under laminar flow conditions (with prior overnight UV radiation); sample tubes were autoclaved and, together with the buffers/solutions used, UV-radiated prior to usage to remove pre-existing DNA. To investigate the effect of the DNA extraction protocol, we also included a set of *n* = 8 human putamen samples (*n* = 6 PD; *n* = 2 HC) that were extracted using the same protocol but without sterile tissue handling (i.e., non-sterile extraction termed PK2). We also had biological controls for the PK2 samples (healthy control brains), but we did not include an analogous negative control group, which is a common approach in other studies.

**Table 1:** Experimental setting

Sample	Sample group	Sample collection	Sample handling	Sample input	DNA extraction
**Human**	OB, *n*= 19 (*n*= 9 PD, *n*= 10 HC)	Non- sterile	Sterile	Brain tissue	Proteinase K
	GFI, *n*= 20 (*n*= 10 PD, *n*= 10 HC)		Sterile		
	PN, *n*= 8 (*n*= 6 PD, *n*= 2 HC)		Non-sterile		
	Cortex, *n*= 3 (SC)	Sterile	Sterile		
**Murine**	SPF, *n*=5 GRF, *n*=3	Non- sterile	Sterile	Brain tissue	Proteinase K
		Sterile			
**Controls**	DNA extraction blank control (KitUKB), *n*= 3	NA	Sterile	Empty	Proteinase K
	Negative control (BF), *n*= 4	NA	Sterile	Tris-HCl buffer	Proteinase K
	Negative control (SW), *n*= 3	NA	Sterile	Sterile water	Proteinase K
	Positive DNA extraction control (mock community), *n*= 6	NA	Sterile	Serially diluted mock community	Proteinase K
	No-template control (KitQIB), *n*= 8	NA	Sterile	PCR reagents	No

*OB* olfactory bulb, *GFI* frontal inferior cortex, *PN* putamen, *SPF* specific pathogen free, *GRF* germ free, *BF* Tris-HCl buffer, *SW* sterile/autoclaved water, *KitUKB* kitome UKB, empty tube, *KitQIB* kitome QIB, PCR reagents, *NA* not applicable

Samples were extracted in a random order. Several types of controls were used to account for contamination: negative controls: *n* = 3 samples consisting of an empty tube (*DNA extraction blank control*), allowing detection of contamination during DNA extraction; *n* = 3 samples with autoclaved water; and *n* = 4 samples with the DNA solving buffer (Tris-HCL) only. Prior to sequencing, *n*=8 samples consisting purely of the sequencing reagents *(no-template control*) were added, allowing detection of contaminant DNA introduced during library preparation and PCR amplification. Negative controls were processed and sequenced alongside the biological samples. Positive controls: *n*= 6 samples of a serial dilution (no dilution, 10-, 100-, 1.000-, 10.000-, and 100.000-fold dilution; similar in design to [17]) of a commercially available microbial community mix (*Zymo mock community*, ZYMO D6300). This community contained eight bacterial and two fungal species of ~1.4 × 10^10^ cells/ml that were precisely characterized and guaranteed to contain < 0.01% contamination. The mock community underwent sterile DNA extraction (PK1) and 16S rRNA gene sequencing (*DNA extraction positive control)* alongside other samples*.*

### DNA extraction

To each 0.5–0.1 mg of frozen tissue sample, 700 μl of proteinase K buffer (10 mMol Tris HCl, 20 mMol EDTA, 150 mMol NaCl), 20 μl of SDS 20%, and 20 μl of fresh proteinase K (20 mg/ml) were added and mixed. After incubation overnight at 55 °C with continuous shaking, 300 μl of saturated aqueous NaCl was added, and samples were incubated on ice for 5 min before centrifugation for 30 min at 14,000 rpm and 4 °C. The resulting supernatant was mixed with 1 ml isopropanol and incubated for 10 min for DNA precipitation. Samples were again centrifuged for 30 min at 14,000 rpm and 4 °C. The pellet was washed twice with 70% ethanol following a centrifugation step, each for 15 min at 14,000 rpm and 4 °C. After drying for 3–5 min, the pellet was resuspended in 50–100 μl Tris-HCl buffer (pH 8.3) prior to storage. DNA extraction revealed A260/280 ratios between 1.7 and 2.0. Samples below 1.7 were discarded.

### 16S rRNA gene sequencing

Genomic DNA was normalized to 5 ng/μl with EB (10mM Tris-HCl). A PCR master mix was made up using 4 μl kapa2G buffer, 0.4 μl dNTP’s, 0.08 μl polymerase, 0.4 μl 10 μM forward-tailed specific primer [18] (Bakt_341F 5′ CCTACGGGNGGCWGCAG), 0.4 μl 10 μM reverse-tailed specific primer [18] (Bakt_805R 5′ GACTACHVGGGTATCTAATCC), and 13.72 μl PCR grade water (from the Kapa2G Robust PCR kit Sigma Catalog No. KK5005) per sample and 19 μl added to each well to be used in a 96-well plate. Specific PCR was run with 95 °C for 5 min, 30 cycles of 95 °C for 30 s, 55 °C for 30 s, and 72 °C for 30 s followed by a final 72 °C for 5 min. Following PCR, a 0.7X SPRI using KAPA Pure Beads (Roche Catalog No. 07983298001) was done eluting the DNA in 20 μl of EB (10 mM Tris-HCl). Following the first PCR and clean-up, a second PCR master mix was made using 4 μl kapa2G buffer, 0.4 μl dNTP’s, 0.08 μl polymerase, and 6.52 μl PCR grade water (from the Kap2G Robust PCR kit) per sample and 11 μl added to each well to be used in a 96-well plate. Two microliters of each P7 and P5 of Nextera XT Index Kit v2 index primers (Illumina Catalogue No. FC-131-2001 to 2004) was added to each well. Finally, 5 μl of clean specific PCR mix was added and mixed. The PCR was run at 95 °C for 5 min, 10 cycles of 95 °C for 30 s, 55 °C for 30s, and 72 °C for 30 s followed by a final 72 °C for 5 min.

Following the PCR reaction, the libraries were quantified using the Quant-iT dsDNA Assay Kit, high sensitivity kit (Catalog No. 10164582), and run on a FLUOstar Optima plate reader. Libraries were pooled following quantification in equal quantities. The final pool was cleaned using 0.7X SPRI using KAPA Pure Beads.

To prepare read sequences for upload to ENA (accession number PRJEB42409), human contaminant reads were removed using kraken2 [19] and following the approach described in [20]. Briefly, reads were classified against the human genome (hg38) and retaining unclassified reads (options --quick --confidence 0.001 were used that might increase false-positive rate, thereby rather removing too many than too few human reads). Reads uploaded to EGA (accession number EGAS00001004757) retained all reads and will be made available upon request.

### Bioinformatic analysis and statistics

Brain samples were sequenced using an Illumina MiSeq in paired end read mode (2×300bp). Before each run, the machine was sterilized and washed using standard Illumina protocols. To analyze raw sequencing data, we used LotuS (http://lotus2.earlham.ac.uk) v. 1.65 [21]. Reads were quality filtered using sdm [20], relying on reads with average quality >27; a binomial estimated at < 2.5 read errors; an accumulated error of < 0.75; sequences that were present less than eight times in a single sample, four times in two samples, or three times in three samples, and the first 170 bp, to build zero-range OTUs [22] (zOTUs). From these clusters, representative sequences were extracted (zOTU seeds) that contained both read pairs and were merged using flash [23], removing chimeric (with uchime3 de novo [24]) and potential PhiX contaminant zOTUs.

These were subsequently aligned to SILVA 138 [25] to determine the taxonomic origin of each zOTU using the least common ancestor algorithm, implemented in LotuS [21]. Further, all reads passing relaxed filtering constraints were mapped onto zOTUs to obtain a zOTU abundance matrix and using the taxonomic assignments, species, genus and family abundance matrices. In the final zOTU abundance, we further removed wrongly barcoded reads using the crosstalk algorithm (med. rate 0.005567) implemented in usearch [26]. This resulted in 3014 zOTUs represented by 3,076,479 reads in the final matrix.

### Additional taxonomic zOTU annotations

To investigate the false-positive assignment of zOTU fasta sequences to bacteria, we tested different standard approaches in the field to assign a taxonomy. Note that this was independent of LotuS taxonomic assignments, as only the zOTU clustered DNA sequences were given to these other approaches. For this, we used the following pipelines [19]: QIIME 1 (v.1.9.1; UCLUST and sortmerna), mothur (v.1.39.5), and Divisive Amplicon Denoising Algorithm 2 (DADA2 v.3.10) [27–29]. Taxonomic comparisons were made based on the Greengenes (v.13.8, clustered at 97% similarity) database [30] for QIIME 1, mothur, and the Ribosomal Database Project (RDP trainset 16/release 11.5) database [31] for dada2. For each classifier, the default parameters were used (qiime1- assign_taxonomy.py: -min_consensus_fraction=: 0.51, --sortmerna_db=None, --sortmerna_e_value=1.0, sortmerna_coverage= 0.9 --sortmerna_best_N_alignments=5, --uclust_max_accepts=3, --similarity=0.9; mothur- classify.seqs function, cut off=: 0.80, ksize=8, iters=100; dada2- assignTaxonomy: minBoot: 50, tryRC=F).

### Off-target amplicon removal

All full length zOTU sequences obtained from LotuS were mapped against the masked hg38 GATK human reference genome (https://gatk.broadinstitute.org/hc/en-us/articles/360035890951-Human-genome-reference-builds-GRCh38-or-hg38-b37-hg19) or the mouse reference genome (https://www.ncbi.nlm.nih.gov/assembly/GCF_000001635.20/). For mapping, minimap2 [32] was used with default parameters. Alignments were filtered to have at least 60% overlap in alignment length to the reference genomes and 200 bp alignment length. Ambiguous quality hits were further controlled by manual blasts.

### Generation of ASV and off-target detection with LotuS-Dada2

To evaluate if dada2 [29]-generated amplicon sequence variants (ASVs) were less prone to cluster off-target amplicons, we used the native dada2 workflow in R, as integrated in LotuS ver 2.00. 5149 ASVs were generated on our dataset with identical options as employed for zOTUs, but “-CL dada2”. Of 5149 ASVs, 521 were detected as chimeric ASVs. Using minimap2, we automatically detected 1763 off-target ASVs (978 human, 785 mouse) at >60% overlap in alignment length.

### Off-target analysis in an independent dataset

To investigate the influence of sequencing primers and datasets, we obtained an additional brain microbiome dataset [4] that contained both ITS and 16S rRNA gene amplicons. The data was accessible online upon request at the European Genome Archive (EGA under the accession numbers: EGAS00001003643 and EGAS00001003644). Using LotuS ver 2.00 to process either with options “-CL unoise” for 16S rRNA gene sequences, we also used minimap2 to detect off-targets matching to the human genome at > 60% overlap in alignment length. Other 16S rRNA gene sequencing studies in human brain tissue were not available online nor upon repeated author requests.

### 16S rRNA gene qPCR

16S rRNA gene copies were quantified by real time PCR (qPCR) using a standard curve method. DNA from a known bacterium was amplified, purified, quantified using QUBIT dsDNA HS assay, and used as a template to generate a standard curve (107 to 102 copies) after serial dilution in salmon sperm using 27F and RP2 primers. Equal volumes of DNA extracted from tissue samples and from the standard curve dilutions were amplified using SYBR Select Master Mix (Thermofisher) in FrameStar 384 Well Skirted PCR Plates (4titute limited). The 16S rRNA gene qPCR results were corrected for the median copy number detected in the DNA extraction blank controls and calculated as per mg tissue and μl sample input, respectively. Assuming a similar per sample distribution as observed in the metabarcoding data, the 16S rRNA gene copy number per sample was then corrected for the percentage of off-target amplifications and contaminant DNA, (Suppl. Table 3). This corrected 16S rRNA gene copy number per sample was used to normalize the metabarcoding data.

### Statistical analysis

Statistical analysis was conducted in R 3.6.1. The zOTU abundance matrix was normalized by (i) dividing each feature by the respective total sample sum and (ii) in a second step by the 16S rRNA gene copy number obtained from qPCR experiments. Sample count matrices were rarefied using the R implementation of the [25] RTK toolkit. Significance between groups of samples was tested with a Kruskal–Wallis test. Intergroup compositional differences were calculated using a PERMANOVA test as implemented in vegan [33] (comparing the intragroup to the intergroup Bray-Curtis distances in a permutation scheme and thus calculating a *P* value). PERMANOVA post hoc *P* values were corrected for multiple testing using the Benjamini–Hochberg false discovery rate (*q* value) [34]. The subsequent statistical analyses were calculated using the R-package vegan with Bray-Curtis distance on the rarefied taxa abundances and visualized with custom R scripts. Sample composition plots were visualized with custom R scripts. Non-metric multidimensional scaling (NMDS) was conducted using the vegan version 2.5-6 R-package based on a Bray-Curtis distance of normalized zOTU abundancies and the function “metaMDS”.

### Post-sequencing contamination removal

The open source R package decontam [35] (https://github.com/benjjneb/decontam) was used to identify contaminants based on two reproducible findings: (i) contaminants appear at higher frequencies in low biomass samples and (ii) are often found in negative controls. Identification of contaminants was achieved using different decontam methods: (i) iC1, *isContaminant* based on prevalence in negative controls, (ii) iC2, *isContaminant* based on frequency in negative controls using absolute 16S rRNA gene copy numbers (inverse correlation of DNA concentration and contaminants), (iii) iC3, *isContaminant* combined prevalence and frequency in negative controls using absolute 16S rRNA gene copy numbers, and (iv) iNC, *isNotcontaminant* based on prevalence in negative controls. All contaminants found with the different decontam methods were removed from further analysis. However, decontam relies on contaminants and true taxa being distinct from each other, an assumption that is affected by cross-contamination, which is not removed by decontam. Therefore, our data was corrected for cross-contamination [36] prior to analysis with decontam.

Taxa already known to be introduced by DNA extraction (Salter et al. [37] and Eisenhofer et al. [16], Suppl. Table 1) were further removed. Finally, we excluded all taxa present in negative controls at an abundance of ≥ 0.01 and all taxa in mock samples not originating from the original biological sample.

## Results

Alongside the *n* = 50 human and *n* = 8 murine brain samples, we also investigated several negative and positive controls for this proof of concept study (see Table 1). Human brain samples included tissues from healthy (healthy control, HC) and PD donors and were comprised of samples from different brain regions (olfactory bulb, inferior frontal gyrus, and putamen), all of which could be affected at different stages of the PD pathology; fresh sterile cortex samples were obtained during resective neurosurgery (sterile cortex, SC). Due to the nature of this very low biomass sequencing experiment, we used special filter and analysing algorithms that are part of the overall study results.

### Quality filtering and removal of off-target amplicons in metabarcoding data

After removal of spurious OTUs by standard 16S rRNA gene sequencing approaches (denoising, de novo chimera removal, crosstalk), we obtained 3014 zero-range OTUs (zOTUs).

A large fraction of zOTUs could not be taxonomically assigned, even to the bacterial phylum level (53.3% of 3014). For this reason, we implemented a filtering algorithm in the LotuS pipeline [21], to map zOTUs onto the human and murine reference genome and determine whether they truly represented bacterial zOTUs. This showed that 1032 (34.2%) of the 3014 zOTUs mapped onto the human or mouse genome. Thus, these were off-target amplifications of the murine or human genome. Notably, these off-targets were not amplicons of human or murine mitochondrial 16S or chromosomal 18S rDNA genes; these would have been taxonomically classified as such using standard reference database such as SILVA [38]. These human or mouse genomic regions had likely some similarity to our primers, as we could detect amplification primers on 1012 of the 1032 off-target amplicons.

Off-target amplicons probably occur due to the extremely low bacterial biomass in samples and primer competition with the dominant host DNA background. Most of the off-target zOTUs identified (887; 86%) had no taxonomic assignment. However, 145 (14%) indeed matched references in the SILVA databases using LotuS least common ancestor (LCA) taxonomic assignments; most of these were assigned to *Firmicutes* (144) with one hit to *Phragmoplastophyta*, which is a plant/eukaryote. The majority of the *Firmicutes* hits could not be classified further while some were matched to *Clostridium difficile* (*n*=38) on species level. These 38 high-quality matches were explained due to highly similar short sequences between the reference 16S rRNA gene and zOTU sequences, often at 100% identity and on average 35 nt length, just passing the default 1e-9 blast e-value confidence threshold used in the LotuS LCA step. Interestingly, these matches occurred at the end of forward reads (average position in merged read was at 275 bp, read length was 300 bp), and we speculate that they could be amplified host DNA regions that were chimeric to bacterial contamination in the sample.

**Fig. 1:**
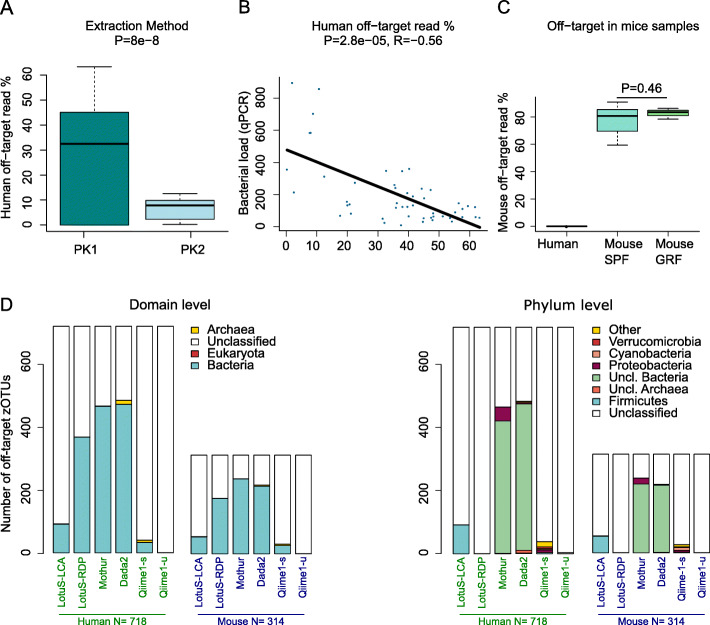
Off-target amplifications dominate amplicon sequences from brain tissue. **a** Clean extraction increases off-target amplifications: The fraction of bacterial reads that could be attributed to off-target amplifications significantly increases in relation to the tissue handling method used (*P*-value = 8e−08, *t*-test, PK2 was more prone to contaminants, see “Methods”). Only samples with > 0.1% off-target relative abundance were included. **b** The relative fraction of off-target reads in each sample is significantly anticorrelated to the amount of 16S rRNA gene copies (determined via qPCR) in each sequencing well (uncorrected, *P* =3e−5, Pearson correlation, rho=− 0.52). **c** The fraction of mouse off-target reads is increased in the 8 mouse brain samples included in this study. No mouse off-target reads were found in human brain samples. The difference in off-target fraction between mice from germ-free facilities (Mouse GRF, *N*=3) was not significantly different to those from mice in specific pathogen-free facilities (Mouse SPF). **d** Classification of off-target amplicons, off-target classification of different databases for taxonomic assignment at the domain or phylum level. Most pipelines misclassify some off-target amplicons as bacteria and give taxonomic assignments at domain and phylum level (shown), in some cases down to the species level (not shown); H, human; M, mouse; Qiime1-s, Qiime 1 sortmerna, Qiime1-u, Qiime 1 uclust

Quantities of bacterial zOTUs corresponding to off-target amplicons varied between samples and seem to be inversely related to the amount of contaminants (Fig. 1 and Suppl. Fig. 1a). We investigated if this was related to the extraction protocols, and indeed the accumulated off-target zOTU abundance in a sample was significantly increased in the PK1 protocol (*P*=8e−8, Fig. 1a) that was less prone to bacterial contamination. Correlating the fraction of off-target zOTU abundance to the amount of 16S rDNA copies found in each sequencing well further confirmed this, being significantly anticorrelated (*P*=3e−5, rho=− 0.52, Fig. 1b). Further, the samples derived from mice held under germ-free conditions, and hypothetically being composed of purely murine DNA, as well as mice from SPF (specific pathogen free) cages, contained approx. 80% murine off-target amplicons (Fig. 1c).

These experiments showed that off-targets are likely the result of missing primer targets, resulting in 16S rRNA gene primers binding to suboptimal binding sites in either mouse or human genomes.

Given the challenge with host-derived off-target amplicons, we examined the performance of other taxonomic assignment algorithms to classify them. Specifically, we compared the taxonomic assignments made by the widely utilized pipelines, QIIME1, Dada2, mothur, and LotuS (Fig. 1d). The RDP classifier [39] used in LotuS-RDP (using by default a confidence threshold of 80) misclassified more off-target amplicons as bacteria at the domain level than the LotuS-LCA approach (Fig. 1d). Fewer sequences were erroneously assigned to bacteria by LotuS-RDP and LotuS-LCA than by mothur and Dada2 (the latter also using the RDP classifier, but with a default confidence threshold of 50). Thus, LotuS-LCA classifies off-target amplicons accurately compared with the other three workflows. QIIME1 (sortmerna and uclust) had the fewest number of false positives, but these represented a wider phylogenetic spectrum.

We next evaluated whether the underlying clustering algorithm had any influence on the detection and classification of off-target amplicons. When generating ASVs instead of zOTUs with Dada2 sequence clustering (Suppl. Table 2), similarly 34.2% of all ASVs (1763 from 5149 ASVs) represent off-target sequences mapping onto the human or the mouse genome. Most of them (1574/89.3%) could not be assigned to a bacterial or eukaryotic SSU sequence, while 10.5% (185 ASVs) were assigned using LotuS’s default taxonomic LCA assignments. Using instead the RDP classifier to assign sequences, 65.7% (759 of 1156) of all off-targets were taxonomically classified to the bacterial domain at high (>0.8) confidence.

Overall, based on our dataset, seemingly all workflows, independent of the underlying sequence clustering (zOTU or ASV), produce false-positive taxonomic assignments by classifying off-target amplicons as bacteria and by assigning a further taxonomic level to them.

Despite their numerous occurrence, off-target amplicons are only one form of false-positive zOTUs that we excluded from our analysis.

### Contaminants in metabarcoding data of human and murine brains

**Fig. 2:**
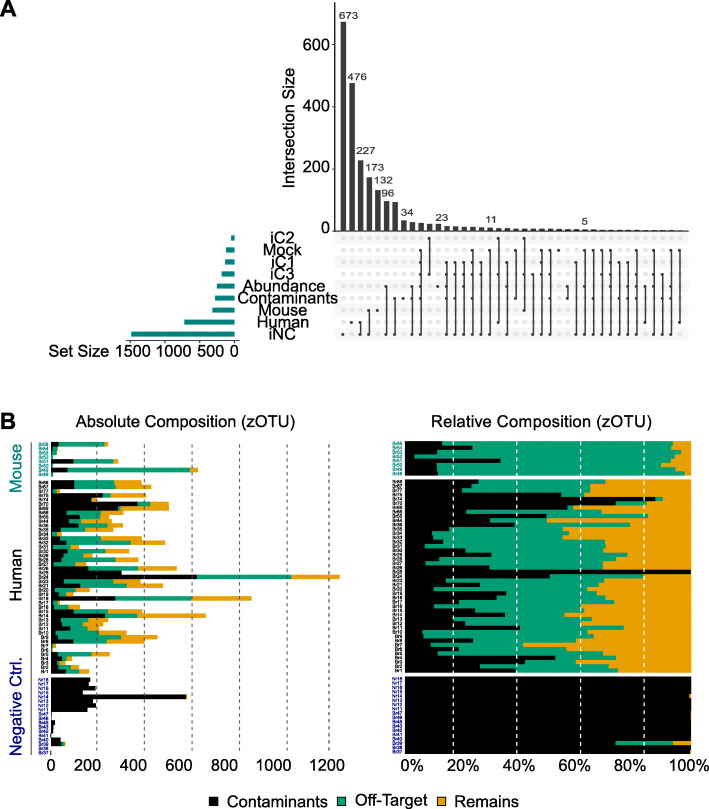
Computational contamination identification. **a** Different approaches used to detect exogenous contamination; iNC, decontam “*isNotcontaminant*”; iC1, decontam “*isContaminant*” based on prevalence in negative controls; iC2, decontam “*isContaminant*” based on frequency in negative controls; iC3, decontam “*isContaminant*” based on combined frequency and prevalence in negative controls; Mouse, off-target zOTUs assigned to the mouse genome; Human, off-target zOTUs assigned to the human genome; Mock, contaminant taxa found in mock community samples; Contaminants, selection of known contaminant taxa in DNA extraction reagents (Suppl. Table 1); Abundance, taxa present in negative controls with relative abundances of ≥ 0.01. Set size represents absolute number of contaminants detected using each method. Intersection size represents the number of uniquely detected zOTUs. All detected contaminants were removed from the further analysis. **b** Relative and absolute abundances of contaminants, off-target amplicons, and remaining zOTUs in all study samples

Having removed off-targets and other false-positive zOTUs (denoised zOTUs, chimeric zOTUs, PhiX matching), we attempted to classify remaining zOTUs (Suppl. Table 3) that might represent true-positive bacteria in brain samples, or contaminant bacteria in reagents, environment, or on technical machines. Two different bioinformatic approaches exist for removing contaminant bacteria, relying either on negative or positive controls [40]. To rigorously identify confounding contaminants, we included both negative and positive controls (Fig. 2a), which were evaluated with different computational methods.

Most contaminant zOTUs were detected using approaches focused on negative controls using decontam [35]. The “i*sNotContaminant*” method was superior to other decontam methods by comparing the prevalence of zOTUs across true samples and negative controls to identify non-contaminants, i.e., detecting contaminants by increased prevalence in negative controls, independently of the absolute bacterial biomass assessed via qPCR. Prevalence-based contaminant identification remains valid even in extremely low biomass samples as it is expected that non-contaminants will appear in larger proportions in true samples than in negative controls. The *isContaminant* method (iC1 and iC2, prevalence or frequency based, respectively) or the combination of both (combined frequency and prevalence in “isContaminant” method, iC3) identified fewer contaminants. Identification of known contaminant taxa introduced through laboratory reagents (Suppl. Table 1) and of contaminants being present in negative controls at an abundance threshold of ≥ 0.01 resulted in comparable findings, removing more contaminants than identified using the positive control, the serially diluted mock community in which the bacterial composition is known a priori. Notably, approximately one third of relative zOTUs abundances in study samples were purely composed of contaminants (Fig. 2b). Analysis of the contaminant taxa (heatmap of contaminants and off-target amplicons, Suppl. Fig. 3) revealed no clear clustering of taxonomic signals in human samples. Non-metric multidimensional scaling (NMDS) of all zOTUs showed a separation of samples based on tissue origin, but after removing off-target and contaminant zOTUs, group cluster was overlapping (Suppl. Figure 4), indicating that initial differences between study groups might be driven by technical signals.

Combining all approaches (contaminant identification and off-target filtering), we thus excluded 2684 zOTUs from further downstream analysis and tested the remaining 331 zOTUs for abundance differences.

### Validation with 16S rRNA gene qPCR results

To quantify the total bacterial biomass in our samples per mg of brain tissue, we used 16S rRNA gene qPCR. 16S rRNA gene copies were corrected for the bacterial biomass expected after both off-target, and contaminant DNA fractions were removed.

To control for our methodological approaches, we also evaluated a set of serially diluted mock samples (positive control) and several negative controls. The number of 16S rRNA gene copies detected in mock samples were in line with the expected qPCR copies given the manufacturer’s bacterial density and our dilutions (Suppl. Fig. 2a) validating our approach for estimating bacterial biomass. However, using amplicon sequencing to determine the mock community composition, we found only six of the eight expected bacterial species at theoretical abundances, but *Listeria monocytogenes* was absent, and *Bacillus subtilis* was at a lower than expected abundance (both bacteria are reported to be difficult to lyse [41, 42]). No mechanical lysis was performed in extracting brain samples or mock community samples to ensure comparability within the analyses. In total, the original taxa composition represented 99.5% of relative zOTU abundance in the undiluted mock community sample and remained stable up to a dilution of 1:10^3^. Contaminant DNA increased with subsequent dilutions. Thus, at the two highest serial dilutions (1:10^4^, 1:10^5^), 13.5% and 18.7% of reads were attributable to contaminants, respectively (Suppl. Fig. 2b).

Negative controls did not differ in terms of the number of 16S rRNA gene copies present (Suppl. Table 4). Overall bacterial biomass was extremely low in biological samples, compared with either the mock samples (115,730,862 16S rRNA gene copies/μl in the undiluted sample, and 4182/μl in the most diluted sample, respectively) or the bacterial biomass found, e.g., in mouse feces (160,000,000.0 (1.6 × 10^8^) 16S rRNA gene copies/mg feces, data from N. Beraza), and we are likely reaching the methodological limits for bacterial detection using 16S rRNA gene qPCR.

**Fig. 3:**
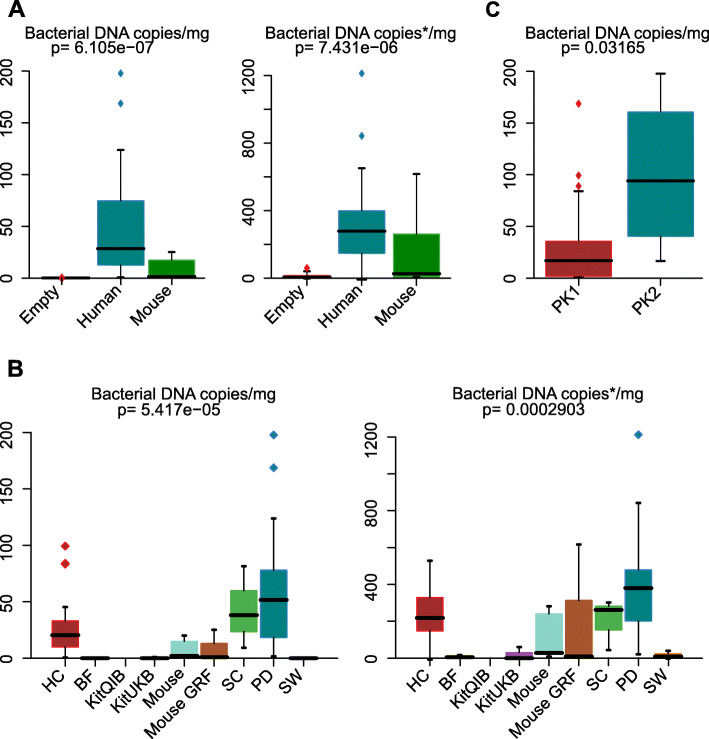
Bacterial DNA assessed using 16S rRNA gene qPCR in study groups. **a** Human samples sign. exceeded mouse and negative controls in terms of 16S rRNA gene copy numbers, most likely resulting from the differentially handled putamen samples (PK2 in C). **b** Bacterial DNA did not differ between human samples (PD, HC, and SC). PD, Parkinson; HC, healthy control; SC, sterile brain from healthy living volunteers. *16S rRNA gene copies post off-target amplicon and contaminant removal

In human brain samples, 16S rRNA gene copy number significantly exceeded the gene copy numbers in mouse samples and negative controls (Fig. 3; Suppl. Table 4), even when correcting for the fraction of off-targets in each sample. This was likely an effect of the differentially handled tissue within the DNA extraction (see below). Although PD samples appeared to contain more bacterial DNA than HC, after off-target correction, there were no significant differences between PD and HC (*P* > 0.05). Furthermore, the quantity of bacterial DNA from SC samples was the same as that from PD or HC samples (*P* > 0.05, Fig. 3b). No correlation was found between the number of 16S rRNA gene copies/quantity of bacterial DNA in human samples and the patient’s age or the post-mortem delay (Suppl. Fig. 2c), making post-mortem bacterial invasion or an age-related effect unlikely.

Last, we investigated the influence of tissue handling during DNA extraction procedure. Non-sterile tissue handling (PK2) resulted in a significantly greater quantity of contaminants in all samples than sterile tissue handling (PK1, Suppl. Fig. 1b). This was also reflected by an increased bacterial biomass assessed with qPCR (Fig. 3c), likely explaining the increased total 16S rRNA gene copy number in human samples.

### Contaminant taxa present in positive and negative controls

In total, 98 different contaminants were present within the mock samples, of which nine had a missing taxonomy on genus level (Suppl. Table 5). As expected, we found no murine genomic DNA in the mock samples. However, in the two most diluted mock samples (dilutions of 1:10^4^ and 1:10^5^), three zOTUs corresponded to human off-target amplicons (Zotu2403 6e-05%, Zotu1715 0.00012%, and Zotu1762 0.000112% relative abundance, respectively, Suppl. Table 5). The taxonomic diversity of contaminants in the negative controls was greatest in the PCR reagents (no template/KitQIB), compared with DNA-extraction blank/KitUKB, DNA buffer, and sterile water (Suppl. Fig. 5). Further analysis showed that 99% of taxa across all negative controls were exogenous contaminants; 1% was human DNA in the DNA extraction blank samples, possibly due to “cross-talk” [26] or human contamination during sequencing.

### Analysis of putative true positive zOTUs in brain samples

**Fig. 4:**
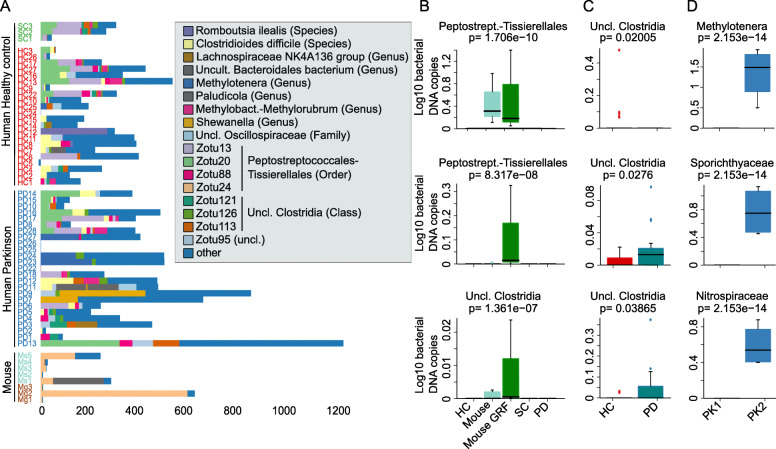
Species composition after contaminant and off-target removal. **a** Final species composition (absolute abundances) after removal of contaminants and off-target amplicons. Abundances were normalized with final 16S rRNA gene copy number and are characterized by an extremely low biomass. Highest biomass was observed in the samples where the DNA was extracted under the PK2 protocol. Mg, mouse; PD, Parkinson; HC, healthy control; SC, sterile brain from healthy living volunteers. **b** The three most sign. enriched zOTUs in study groups after automatic off-target amplicon removal. In-depth checks with manual Blast revealed that these most abundant zOTUs mapped to the human or the mouse genome or were likely contaminant taxa (see text)

After correction for off-target zOTUs and contaminants, we further tested the abundance of zOTUs for putative differences among study groups. The filtered zOTU composition was extremely low in species abundances and irregularly distributed among all biological samples (Fig. 4a); most remaining zOTUs were unclassified at species level.

All taxonomic signals of true positive zOTUs (Fig. 4a) were reassessed using a manual Blast (Suppl. Table 6). Six out of the 17 most abundant zOTUs across all study samples belonged to either the human or the mouse genome (zOTU20/uncl. Peptostreptococcales-Tissierellales, zOTU24/uncl. Peptostreptococcales-Tissierellales, zOTU56/Clostridioides difficile, zOTU88/uncl. Peptostreptococcales-Tissierellales, zOTU95/unlcassified, and zOTU126/uncl. Clostridia); three were either classified as bacteria or human/mouse genome with similar query coverage and percent identity (zOTU13/uncl. Peptostreptococcales-Tissierellales, zOTU121/uncl. Clostridia, and zOTU113/uncl. Clostridia); and one was classified as an uncultured organism (zOTU180/uncl. Oscillospiraceae). However, six were accurately classified as bacteria (of which five were classified as uncultured bacteria in the NCBI databases with manual Blast; zOTU34/uncl. Methylotenera, zOTU93/uncultured Bacteroidales bacterium, zOTU763/Romboutsia ilealis, zOTU217/uncl. Shewanella, zOTU91/uncl. Lachnospiraceae NK4A136 group, and zOTU254/uncl. Paludicola), occurring across all types of study samples including negative controls. One zOTU (zOTU124), assigned to Methylobacterium-Methylorubrum by LotuS and manual Blast, was present only in human samples (*n* = 11, across PD, SC, and HC), and at extremely low abundances. These bacteria are commonly found in the atmosphere, soil, and on human skin, but also in laboratory reagents [43, 44]. Very rarely, they have been reported as opportunistic pathogens in clinical samples [44]. Thus, there is a remote possibility that these might represent undetected invasions of healthy and diseased brains, but due to the pathogenic nature and the frequent presence in soil and reagents, it seems unlikely that they are true positives. Further, plotting the frequency of zOTU124 in relation to bacterial biomass showed an inverse relationship (Suppl. Fig. 6), as would be expected for a contaminant bacterium.

Of the remaining zOTUs that appeared to be true positive bacteria in human samples, 52 were uniquely present in putamen samples; the five most abundant were zOTU34/Methylotenera, zOTU187/Sporichthyaceae, zOTU231/Nitrospiraceae, zOTU259/Moraxellaceae bacterium HYN0046, and zOTU256/Comamonadaceae (Fig. 4d), which were environmental bacteria in soils and waste water or present as pathogens in the oral human cavity. However, it is likely that this was as a result of “non-sterile” (PK2) tissue handling during DNA extraction, which only became apparent after statistical blocking for tissue handling type. The zOTUs from putamen samples all matched known bacteria more closely than host genomes and would therefore seem to represent additional contaminants in our extraction protocols (Suppl. Fig. 1b). These contaminants were likely missed with our initial computational approach, as we did not include negative controls processed with the same PK2 protocol, more prone to contaminants (see above).

Manual Blast analysis of enriched zOTUs in the olfactory bulb (not shown) revealed them all to be of human origin, except zOTU2986/*Salmonella enterica* which was identified as an uncultured bacterium clone. Since zOTU2986 was also present (at low prevalence) in human and murine brain samples, and in negative controls, it appears almost certainly to be a false-positive.

Further, in human samples, six zOTUs contributed to the significant difference between PD and HC samples: zOTU643, zOTU1896, zOTU873, zOTU607, zOTU939, and zOTU1931 (Fig. 4c). These six were all assigned to *Clostridia* by LotuS-LCA and appeared to be of human origin when using a manual Blast search. Thus, they represent additional off-target amplicons not detected in our automatic off-target removal.

The same was true for five enriched zOTUs from murine samples (Fig. 4b), of which four (zOTU24, zOTU593, zOTU590, and zOTU1665) were originally assigned to *Clostridia* but were ultimately found to be murine genomic DNA. Manual Blast identified ZOTU847/uncl. Lachnospiraceae NK4A136 group, which was enriched in murine samples, as an uncultured bacterium clone, but it was only present in two samples.

An additional 75 zOTUs that remained in the analysis after automatic off-target removal and automatic contamination removal were discovered to be off-target amplicons through manual blast searches. All other workflows evaluated also classified the majority of these remaining 75 zOTUs as bacteria (Suppl. Fig. 7). Thus, all the in-depth evaluations of brain-enriched microbes in our study (with one very unlikely exception) can be considered as false-positive zOTUs, and no convincing taxonomic signal of a consistent bacterial colonization/presence was found.

### Validation of study results in an independent dataset

In order to assess whether off-target amplifications could emerge as a broader phenomenon in low-biomass 16S rRNA gene sequencing experiments, we additionally evaluated an independent dataset (a 16S rRNA gene sequencing experiment in human brain tissue from PD and healthy individuals, see “Methods”).

Of 2165 generated zOTUs in this dataset, 8.7% (198 zOTUs) were mapping onto the human genome. After filtering zOTUs with no taxonomic assignment and technical artifacts, we obtained 1264 zOTUs, which could putatively originate from bacteria. As positive or negative controls were not available in this dataset, bioinformatic approaches to remove contaminants were inapplicable. However, when matching the detected species to typical contaminants (coming from laboratory reagents, Suppl. Table 1), 402 zOTUs matched to known contaminant bacteria.

## Discussion

Previous studies have suggested the existence of a human “brain microbiome” that might be associated with age-related neurodegenerative diseases, like PD (or AD). To determine whether this is the case, we undertook a low-biomass optimized 16S rRNA gene sequencing study in brain tissues of healthy and PD-affected individuals, using both experimental and bioinformatic contamination controls.

### Reports of putative bacteria in the brain

Although numerous microorganisms can invade the bloodstream, very few bacteria can spread to the meninges or the brain parenchyma due to the blood-brain-barrier, which protects the CNS from infection.

Pathogenic extracellular bacteria invade the CNS by interacting directly with the CNS barrier, whereas intracellular bacteria enter the brain by spreading into host peripheral immune cells [45]. Mono-microbial infections are relatively easy to detect because of the acute severe illnesses they cause (e.g., as meningococcal meningitis). In contrast, polymicrobial (bacterial or fungal) “infections” have been repeatedly reported in the brain tissue of patients with various chronic, neurodegenerative diseases [5–7, 9] or neuro-immunological diseases [10]. One 16S rRNA gene sequencing study [7] of brain tissue from AD patients reported a 5- to 10-fold increase in total bacterial reads in AD patients compared with controls; however, bacterial reads obtained from 16S rRNA gene sequencing do not accurately reflect biological differences but technical variations during library preparation. Furthermore, fungal and bacterial species have been detected together in the brain tissue of patients with AD [5, 6], ALS [9], and multiple sclerosis [10] using immunohistochemistry, nested PCR, or 16S rRNA gene sequencing techniques. However, although some of the experimental protocols were performed under sterile conditions virtually, all studies lacked adequate negative (or positive) controls and did not appropriately account for exogenously introduced DNA. Most taxa reported in these studies are also known contaminants (Actinobacteria or Proteobacteria; Suppl. Table 1) that could have been introduced during the experimental process rather than originating from the tissue itself.

Previously, it was hypothesized that peripheral blood was the source of Proteobacteria or Actinobacteria in brain tissue, as blood from control patients also contained a major proportion of these taxa [46]. However, although the authors of this study tested their reagents to minimize contamination, no reports on negative/blank controls were made, nor were computational contamination removal techniques used. In another study, the authors used two different DNA extraction protocols for extracting brain samples from two independent AD cohorts [8], but the presence of exogenous DNA contamination could not be determined missing adequate controls.

Several of these studies [4, 7, 9] have reported OTUs but only at the phylum or family levels, reflecting inaccurate bioinformatics or taxa that are not represented in databases. Further, *Cyanobacteria* and *Gemmatimonadete*s have been found in brain tissue of ALS and PD patients [4, 8, 9], but are unlikely to be host associated because their origins are to our best knowledge aqueous and from soils, respectively. Aside from bacteria, on several occasions fungal, species have been detected in CNS tissues, both intra- and extracellular, using either ITS amplicon sequencing or immunohistochemistry [5, 6, 47, 48], a hypothesis that was not addressed in this work, relying entirely on 16S rRNA gene sequencing of bacteria.

### Off-target amplicons and exogenous contaminants in low biomass sequencing

In our analysis of low-biomass brain tissue samples, we were confronted with several independent sources of false positive OTUs, each of which could have led to misinterpretations.

The degree by which samples are contaminated by exogenous DNA is inversely proportional to the starting bacterial biomass of the sample and therefore extremely low biomass samples are most affected [16, 37, 49]. Importantly, in our study, 54.8% of zOTUs could be attributed to exogenous DNA contamination, which is in line with the findings from other studies of low biomass samples, such as samples from the lower airways (10–50% contaminants) [50]. Contamination can arise at any stage of the experimental procedures and can include “cross-contamination” by DNA from barcodes or amplicons from neighbouring wells or tubes, and from the investigators themselves [16, 37].

Following recent literature [16, 37], we used several approaches to avoid and/or reduce pre- and post-sequencing contamination, i.e., inclusion of suitable negative and positive controls, and specialized computational approaches in bioinformatic processing. We could not find a true taxonomic signal that would confirm bacteria resident in either healthy or PD individuals’ brains. Even in the olfactory bulb, a previously suggested entry point for bacteria, no bacterial signal could be detected.

Furthermore, we identified a large proportion of off-target amplicons from the host genome, representing 34.2% of all zOTUs found in our dataset. Off-target amplicons are different from exogenous contamination because their occurrence in biological samples can be easily misinterpreted as a true biological signal: they do not occur in negative controls and are therefore not detected by standard contamination removal algorithms. Also, reanalyzing an independent brain-derived 16S rRNA gene sequencing dataset with different primers used, we similarly found off-target amplicons. We further found the occurrence of off-target amplicons to negatively correlate with abundance of bacteria in our samples, indicating that off-target amplicons become a more prevalent confounder in extreme low-bacterial/high-host biomass scenarios—such as proving the presence of bacteria in actually sterile tissue.

It is important to make a distinction between off-target amplicons and 18S rRNA or mitochondrial 16S OTU’s, as these are actually on-target (but often unwanted). The latter are easily correctly classified in most pipelines as mitochondrial and eukaryotic rRNA gene sequences, as standard reference ribosomal SSU databases will include these sequences (e.g., SILVA [51]). Our reanalysis of taxonomic assignments showed that all tested analysis workflows (LotuS-LCA, LotuS-RDP, Quiime1, mothur, and Dada2) taxonomically misclassified a proportion of off-target zOTUs as bacteria. This was also the case when using a different sequence clustering algorithm to generate ASVs with dada2, suggesting a generalizable problem.

## Conclusions

It is reasonable and appealing to hypothesize that a bacterial invasion into the CNS could contribute to the immune responses observed in neurodegenerative disorders such as PD (or AD). However, in our study based on 16S rRNA gene sequencing, we found no valid taxonomic signals and therefore no convincing evidence to support the idea that there could be either bacterial infection/invasion of PD brains and olfactory bulb, or bacteria residing in the brains of healthy humans.

Our study highlights the importance of extensive controls and bioinformatic tools to avoid false positive signals caused by either exogenous DNA contamination or what we describe here as off-target amplicons, a class of confounding factors that can easily be misinterpreted as biological signals.

## Supplementary Information

**Additional file 1: Suppl. Figure 1.** Off target amplicons and contaminants in study samples. A: Off target amplicons were most abundant in murine samples. B: PK2 DNA extraction (contaminant prone) in putamen samples resulted in significantly more contaminants compared with PK1 DNA extraction protocol.

**Additional file 2: Suppl. Figure 2.** Mock community and 16S rRNA gene copy number correlations. A: Log16S rRNA gene copy number in mock samples corresponding to the theoretical values. The theoretical values were calculated based on the cell concentration given by the manufacturer (~1.4 x 10^10^ cells/ml), measured values are corrected for DNA extraction blank controls and are in line with the theoretical values. B: Log_10_ 16S rRNA gene copies (bars) in relation to the ASV abundances (on top of bars) in mock samples. Note that the original mock community composition remained stable up to a dilution of 10^3^. C: Log_10_ 16S rRNA gene copy number does not correlate significantly with age of donor or post mortem delay (PMD).

**Additional file 3: Suppl. Figure 3.** Heatmap of removed contaminants. Heatmap of the relative abundance of zOTUs (contaminants and off-target amplicons) that were removed from downstream analysis. #: contaminant categories, number of computational contamination removal approaches detecting a respective zOTU, Heatscale represents relative zOTU abundances in samples.

**Additional file 4: Suppl. Figure 4.** Non-metric multidimensional scaling (NMDS). NMDS ordination of study groups (A) before any, (B) after automatic contamination and off-target removal, and (C) after removal of additional off-target zOTUs (N=75, Suppl. Fig. 7). Distance between study groups diminishes after computational removal approaches. Note that additional off-targets and contaminants later described in the text and Suppl. Fig. 7 are still included in B. Symbols represent samples, and distances between symbols represent similarities, i.e. closer symbols are more similar than distant symbols. Human, all human samples; Mouse, all murine samples; Empty, all negative controls; Mock, mock community samples.

**Additional file 5: Suppl. Figure 5.** Negative controls. Taxonomic composition (genus level) of negative controls normalized by 16S rRNA gene copy number; further analysis showed 99% of all zOTUs in negative control samples were attributable to contaminant bacteria, while <1% represented human DNA (only present in KitomeUKB/DNA extraction blank control, possibly representing cross-talk or human contaminants). Most taxa were introduced during the sequencing process, thus, library preparation and PCR amplification (KitomeQIB/no template control). Each square represents one 16S rRNA gene copy/μl. KitomeUKB, negative control of reagents used in the UKB, Bonn, Germany. KitomeQIB, negative control of reagents used in the QIB, Norwich, UK.

**Additional file 6: Suppl. Figure 6.** Frequency of zOTU124 in relation to the bacterial biomass assessed with 16S rRNA gene qPCR strengthens interpretation of contamination origin.

**Additional file 7: Suppl. Figure 7.** Classification of remaining and manually re-analysed zOTUs. The 75 zOTUs that remained in the analysis after automatic off-target removal and automatic contamination removal were discovered to be off-target amplicons through manual blast searches. These 75 zOTUs were again misclassified as bacterial phyla by most amplicon pipelines; note that only zOTUs are shown, which had already been processed using the LotuS-LCA approach, thus the majority of unclassified zOTUs had already been excluded. H, human; M, mouse; Qiime1-s, Qiime1 sortmerna, Qiime1-u, Qiime1 uclust; ?, unclassified.

**Additional file 8: Suppl. Table 1.** Published contaminants from ‘blank controls’ (according to Salter et al .[37] and Eisenhofer et al., 2019 [16]

**Additional file 9: Suppl. Table 2.** Taxonomic classification of off-target amplicons based on ASV clustering. Classification is either based on LotuS-LCA or LotuS-RDP.

**Additional file 10: Suppl. Table 3.** Overview of different classes of contaminants identified and corrected for bioinformatically.

**Additional file 11: Suppl. Table 4.** qPCR-derived 16S rRNA gene copies in brain tissue and control samples. PD, Parkinson’s disease, HC, healthy control; SC, sterile control; GRF, germ free; SPF, specific pathogen free; KitUKB, empty tube; BF, Tris-HCl buffer; KitQIB, PCR reagents; SW, sterile/autoclaved water; NA, not applicable; Data is presented as the median. P-values are given for the final 16S rRNA gene copy numbers; **P=3.15x10-6 vs. controls; P=0.0018 vs. mouse; *P=0.0014 vs controls.

**Additional file 12: Suppl. Table 5.** Analysis of off-target amplicons in mock samples.

**Additional file 13: Suppl. Table 6.** In depth check of putative true bacteria in brain samples with manual Blast n.

## Data Availability

The datasets generated and analyzed as part of the current study are publicly available in the ENA repository (https://www.ebi.ac.uk/ena) under the accession number PRJEB42409. Author’s note: as the dataset contains a large proportion of human genomic information, human genomic sequences were removed from the publicly available dataset; the whole dataset including human genomic sequences is available in the EGA repository upon author’s request (https://www.ebi.ac.uk/ega) under the accession number EGAS00001004757. R analysis scripts are available on https://github.com/hildebra/brainMicrobiomeRscripts. Changes to the LotuS pipeline to reliably detect off-targets are implemented in LotuS2 at http://lotus2.earlham.ac.uk/.

## References

[1] Spielman LJ, Gssibson DL, Klegeris A (2018). Unhealthy gut, unhealthy brain: the role of the intestinal microbiota in neurodegenerative diseases. Neurochem Int..

[2] Bedarf JR, Hildebrand F, Goeser F, Bork P, Wüllner U (2019). Das Darmmikrobiom bei der Parkinson-Krankheit. Der Nervenarzt..

[3] Bedarf JR, Hildebrand F, Coelho LP, Sunagawa S, Bahram M, Goeser F, et al. Functional implications of microbial and viral gut metagenome changes in early stage L-DOPA-naïve Parkinson’s disease patients. Genome Med. 2017;9:39. 10.1186/s13073-017-0428-y. 10.1186/s13073-017-0428-y PMC5408370 28449715

[4] Pisa D, Alonso R, Carrasco L (2020). Parkinson’s disease: a comprehensive analysis of fungi and bacteria in brain tissue. Int J Biol Sci..

[5] Alonso R, Pisa D, Fernández-Fernández AM, Carrasco L. Infection of fungi and bacteria in brain tissue from elderly persons and patients with Alzheimer’s disease. Front Aging Neurosc. 2018;10. 10.3389/fnagi.2018.00159. 10.3389/fnagi.2018.00159 PMC5976758 29881346

[6] Pisa D, Alonso R, Fernández-Fernández AM, Rábano A, Carrasco L (2017). Polymicrobial infections in brain tissue from Alzheimer’s disease patients. Sci Rep..

[7] Emery DC, Shoemark DK, Batstone TE, Waterfall CM, Coghill JA, Cerajewska TL, et al. 16S rRNA next generation sequencing analysis shows bacteria in Alzheimer’s post-mortem brain. Front Aging Neurosci. 2017;9. 10.3389/fnagi.2017.00195. 10.3389/fnagi.2017.00195 PMC5476743 28676754

[8] Westfall S, Dinh DM, Pasinetti GM. Investigation of potential brain microbiome in Alzheimer’s disease: implications of study bias. J Alzheimers Dis. 2020:1–12. 10.3233/JAD-191328. 10.3233/JAD-191328 32310171

[9] Alonso R, Pisa D, Carrasco L. Searching for bacteria in neural tissue from amyotrophic lateral sclerosis. Front Neurosci. 2019;13. 10.3389/fnins.2019.00171. 10.3389/fnins.2019.00171 PMC6399391 30863279

[10] Alonso R, Fernández-Fernández AM, Pisa D, Carrasco L (2018). Multiple sclerosis and mixed microbial infections. Direct identification of fungi and bacteria in nervous tissue. Neurobiol Dis..

[11] Servick K. Do gut bacteria make a second home in our brains? Science. 2018. 10.1126/science.aaw0147.

[12] Qian Y, Yang X, Xu S, Wu C, Qin N, Chen S-D, et al. Detection of microbial 16S rRNA gene in the blood of patients with Parkinson’s disease. Front Aging Neurosci. 2018;10. 10.3389/fnagi.2018.00156. 10.3389/fnagi.2018.00156 PMC5976788 29881345

[13] Isaacs-Ten A, Echeandia M, Moreno-Gonzalez M, Brion A, Goldson A, Philo M, et al. Intestinal microbiome-macrophage crosstalk contributes to cholestatic liver disease by promoting intestinal permeability. Hepatology. 2020:hep.31228. 10.1002/hep.31228. 10.1002/hep.31228 PMC7839474 32168395

[14] Rey NL, Wesson DW, Brundin P (2018). The olfactory bulb as the entry site for prion-like propagation in neurodegenerative diseases. Neurobiol Dis..

[15] Braak H, Del Tredici K, Rüb U, de Vos RAI, Jansen Steur ENH, Braak E (2003). Staging of brain pathology related to sporadic Parkinson’s disease. Neurobiol Aging.

[16] Eisenhofer R, Minich JJ, Marotz C, Cooper A, Knight R, Weyrich LS (2019). Contamination in low microbial biomass microbiome studies: issues and recommendations. Trends Microbiol..

[17] Karstens L, Asquith M, Davin S, Fair D, Gregory WT, Wolfe AJ, et al. Controlling for contaminants in low-biomass 16S rRNA gene sequencing experiments. mSystems. 2019;4. 10.1128/mSystems.00290-19. 10.1128/mSystems.00290-19 PMC6550369 31164452

[18] Klindworth A, Pruesse E, Schweer T, Peplies J, Quast C, Horn M (2013). Evaluation of general 16S ribosomal RNA gene PCR primers for classical and next-generation sequencing-based diversity studies. Nucleic Acids Res..

[19] Wood DE, Lu J, Langmead B. Improved metagenomic analysis with Kraken 2. Genome Biol. 2019;20:257. 10.1186/s13059-019-1891-0. 10.1186/s13059-019-1891-0 PMC6883579 31779668

[20] Hildebrand F, Moitinho-Silva L, Blasche S, Jahn MT, Gossmann TI, Huerta-Cepas J (2019). Antibiotics-induced monodominance of a novel gut bacterial order. Gut..

[21] Hildebrand F, Tadeo R, Voigt A, Bork P, Raes J (2014). LotuS: an efficient and user-friendly OTU processing pipeline. Microbiome..

[22] Edgar RC. UNOISE2: improved error-correction for Illumina 16S and ITS amplicon sequencing. 10.1101/081257.

[23] Magoc T, Salzberg SL (2011). FLASH: fast length adjustment of short reads to improve genome assemblies. Bioinformatics..

[24] Edgar RC. UCHIME2: improved chimera prediction for amplicon sequencing. 10.1101/074252.

[25] Saary P, Forslund K, Bork P, Hildebrand F (2017). RTK: efficient rarefaction analysis of large datasets. Bioinformatics..

[26] Edgar RC. UNCROSS2: identification of cross-talk in 16S rRNA OTU tables. bioRxiv. 2018;01:01.400762. 10.1101/400762.

[27] Caporaso JG, Kuczynski J, Stombaugh J, Bittinger K, Bushman FD, Costello EK (2010). QIIME allows analysis of high-throughput community sequencing data. Nature Methods..

[28] Schloss PD, Westcott SL, Ryabin T, Hall JR, Hartmann M, Hollister EB (2009). Introducing mothur: open-source, platform-independent, community-supported software for describing and comparing microbial communities. Appl Environ Microbiol..

[29] Callahan BJ, McMurdie PJ, Rosen MJ, Han AW, Johnson AJA, Holmes SP (2016). DADA2: high-resolution sample inference from Illumina amplicon data. Nat Methods..

[30] DeSantis TZ, Hugenholtz P, Larsen N, Rojas M, Brodie EL, Keller K (2006). Greengenes, a chimera-checked 16S rRNA gene database and workbench compatible with ARB. Appl Environ Microbiol.

[31] Cole JR, Wang Q, Fish JA, Chai B, McGarrell DM, Sun Y (2014). Ribosomal database project: data and tools for high throughput rRNA analysis. Nucleic Acids Res..

[32] Li H (2018). Minimap2: pairwise alignment for nucleotide sequences. Bioinformatics..

[33] ANDERSON MJ (2008). A new method for non-parametric multivariate analysis of variance. Austral Ecol..

[34] Benjamini Y, Hochberg Y (1995). Controlling the false discovery rate: a practical and powerful approach to multiple testing. J Royal Stat Soc Series B (Methodological)..

[35] Davis NM, Proctor DM, Holmes SP, Relman DA, Callahan BJ (2018). Simple statistical identification and removal of contaminant sequences in marker-gene and metagenomics data. Microbiome..

[36] Wright ES, Vetsigian KH (2016). Quality filtering of Illumina index reads mitigates sample cross-talk. BMC Genomics..

[37] Salter SJ, Cox MJ, Turek EM, Calus ST, Cookson WO, Moffatt MF, et al. Reagent and laboratory contamination can critically impact sequence-based microbiome analyses. BMC Biol. 2014;12:87. 10.1186/s12915-014-0087-z. 10.1186/s12915-014-0087-z PMC4228153 25387460

[38] Yilmaz P, Parfrey LW, Yarza P, Gerken J, Pruesse E, Quast C (2014). The SILVA and “All-species Living Tree Project (LTP)” taxonomic frameworks. Nucleic Acids Res..

[39] Wang Q, Garrity GM, Tiedje JM, Cole JR (2007). Naïve Bayesian classifier for rapid assignment of rRNA sequences into the new bacterial taxonomy. Appl Environ Microbiol..

[40] Ducarmon QR, Hornung BVH, Geelen AR, Kuijper EJ, Zwittink RD. Toward Standards in clinical microbiota studies: comparison of three DNA extraction methods and two bioinformatic pipelines. mSystems. 2020;5. 10.1128/mSystems.00547-19. 10.1128/mSystems.00547-19 PMC7018525 32047058

[41] Vandeventer PE, Weigel KM, Salazar J, Erwin B, Irvine B, Doebler R (2011). Mechanical disruption of lysis-resistant bacterial cells by use of a miniature, low-power, disposable device. J Clin Microbiol.

[42] Aasta Agersborg RDIM (1997). Sample preparation and DNA extraction procedures for polymerase chain reaction identification of Listeria monocytogenes in seafoods. Int J Food Microbiol..

[43] Green PN, Ardley JK (2018). Review of the genus Methylobacterium and closely related organisms: a proposal that some Methylobacterium species be reclassified into a new genus, Methylorubrum gen. nov. Int J Syst Evol Microbiol.

[44] Boden R. Methylotrophy, the human body, and disease. In: Goldfine H, editor. Health consequences of microbial interactions with hydrocarbons, oils, and lipids. Handbook of Hydrocarbon and Lipid Microbiology. Heidelberg: Spinger; 2019.

[45] Coureuil M, Lécuyer H, Bourdoulous S (2017). Nassif X. A journey into the brain: insight into how bacterial pathogens cross blood–brain barriers. Nature Reviews. Microbiology..

[46] Païssé S, Valle C, Servant F, Courtney M, Burcelin R, Amar J (2016). Comprehensive description of blood microbiome from healthy donors assessed by 16S targeted metagenomic sequencing. Transfusion..

[47] Pisa D, Alonso R, Rábano A, Rodal I, Carrasco L (2015). Different brain regions are infected with fungi in Alzheimer’s disease. Sci Rep..

[48] Parady B (2018). Innate immune and fungal model of Alzheimer’s disease. J Alzheimers Dis Rep..

[49] Glassing A, Dowd SE, Galandiuk S, Davis B, Chiodini RJ. Inherent bacterial DNA contamination of extraction and sequencing reagents may affect interpretation of microbiota in low bacterial biomass samples. Gut Pathogens. 2016;8:24. 10.1186/s13099-016-0103-7. 10.1186/s13099-016-0103-7 PMC4882852 27239228

[50] Drengenes C, Wiker HG, Kalananthan T, Nordeide E, Eagan TML, Nielsen R (2019). Laboratory contamination in airway microbiome studies. BMC Microbiol..

[51] Quast C, Pruesse E, Yilmaz P, Gerken J, Schweer T, Yarza P (2012). The SILVA ribosomal RNA gene database project: improved data processing and web-based tools. Nucleic Acids Res..

