# Supplementary figures and images for: Much ado about nothing? Off-target amplification can lead to false-positive bacterial brain microbiome detection in healthy and Parkinson’s disease individuals
Source: Microbiome. 2021 Mar 26;9:75. doi: 10.1186/s40168-021-01012-1 (PMC8004470; doi:10.1186/s40168-021-01012-1)

**A**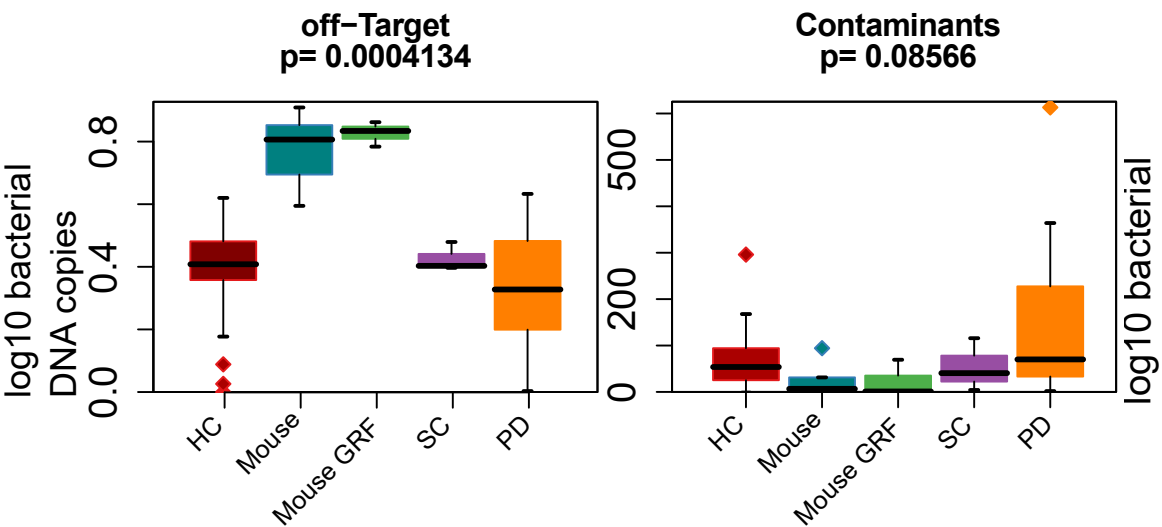**B**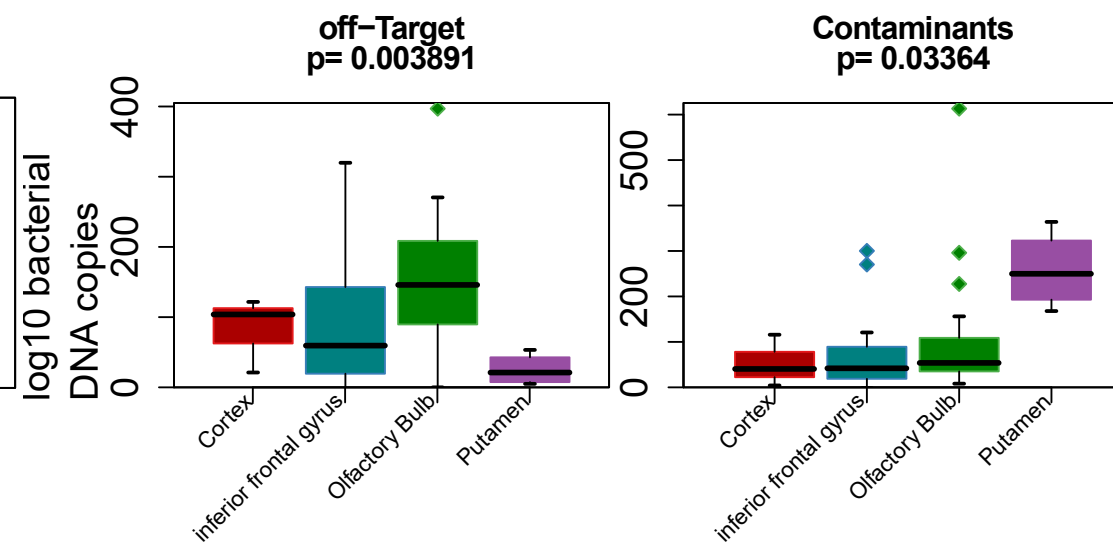**C**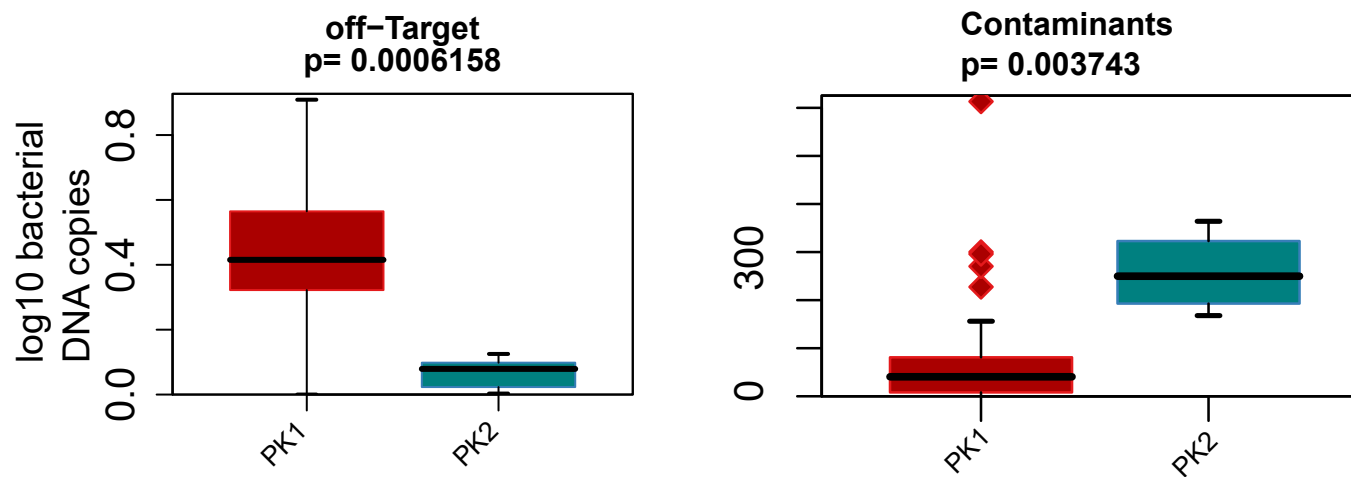

Supplement: Supplementary file 2 — Additional file 1: Suppl. Figure 1. Off target amplicons and contaminants in study samples. A: Off target amplicons were most abundant in murine samples. B: PK2 DNA extraction (contaminant prone) in putamen samples resulted in significantly more contaminants compared with PK1 DNA extraction protocol. [file 40168_2021_1012_MOESM2_ESM.pdf]

A

| Dilution (Mock) | Theoretic log bact. DNA copies | Measured log bact. DNA copies |
|-----------------|--------------------------------|-------------------------------|
| no              | 8.69                           | 8.06                          |
| 1:10            | 7.69                           | 7.54                          |
| 1:100           | 6.69                           | 6.29                          |
| 1:1.000         | 5.69                           | 5.00                          |
| 1:10.000        | 4.69                           | 4.42                          |
| 1:100.000       | 3.69                           | 3.62                          |

B

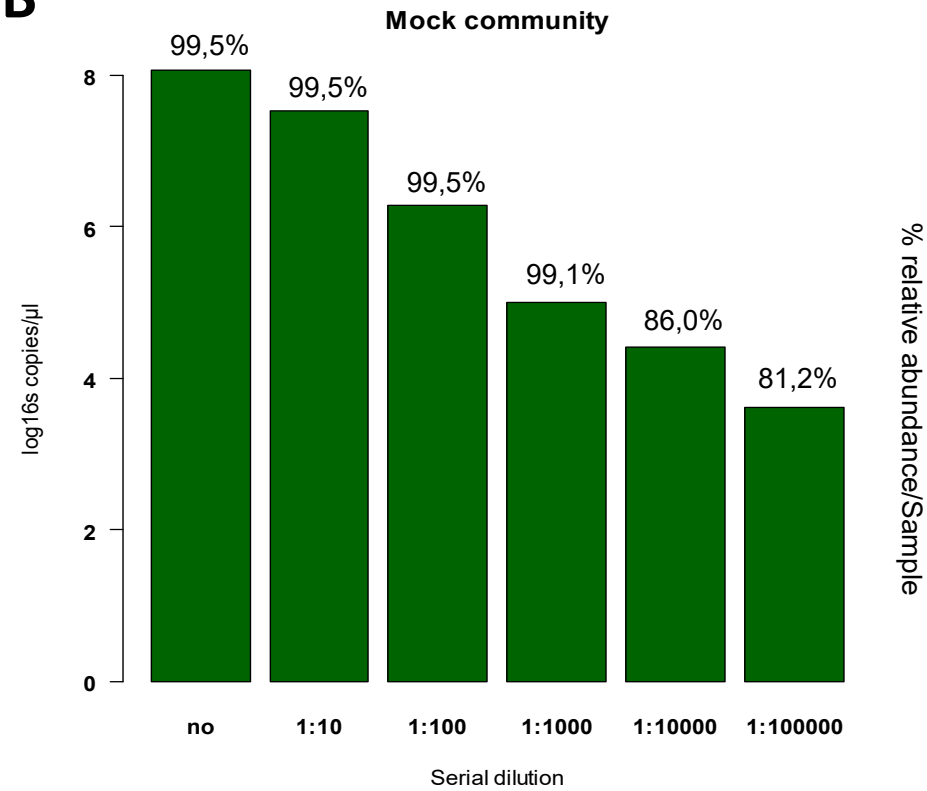

C

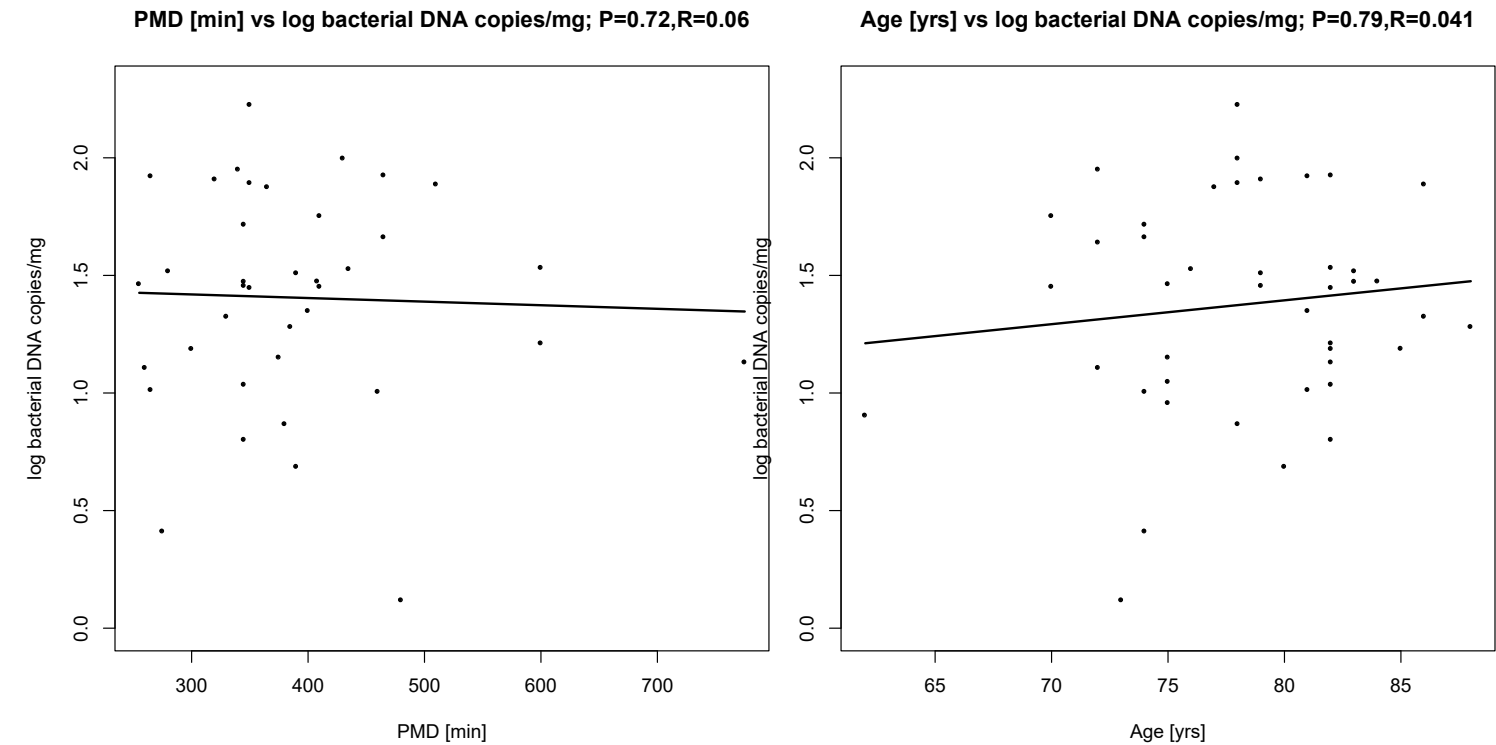

Supplement: Supplementary file 3 — Additional file 2: Suppl. Figure 2. Mock community and 16S rRNA gene copy number correlations. A: Log16S rRNA gene copy number in mock samples corresponding to the theoretical values. The theoretical values were calculated based on the cell concentration given by the manufacturer (~1.4 x 10^10^ cells/ml), measured values are corrected for DNA extraction blank controls and are in line with the theoretical values. B: Log10 16S rRNA gene copies (bars) in relation to the ASV abundances (on top of bars) in mock samples. Note that the original mock community composition remained stable up to a dilution of 103. C: Log10 16S rRNA gene copy number does not correlate significantly with age of donor or post mortem delay (PMD). [file 40168_2021_1012_MOESM3_ESM.pdf]

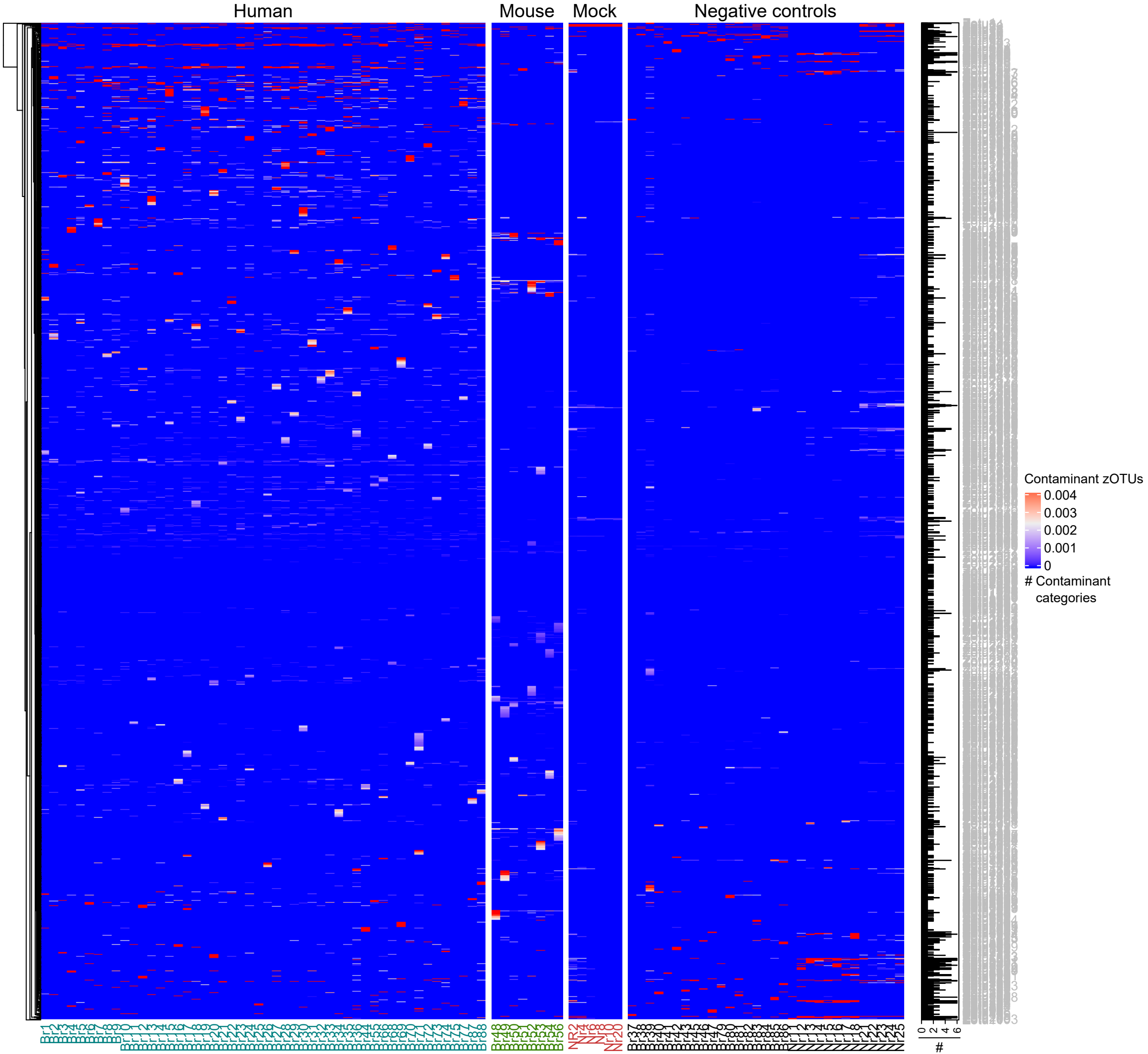

Supplement: Supplementary file 4 — Additional file 3: Suppl. Figure 3. Heatmap of removed contaminants. Heatmap of the relative abundance of zOTUs (contaminants and off-target amplicons) that were removed from downstream analysis. #: contaminant categories, number of computational contamination removal approaches detecting a respective zOTU, Heatscale represents relative zOTU abundances in samples. [file 40168_2021_1012_MOESM4_ESM.pdf]

**A**

Triplot - 2D NMDS - Dim 1 &amp; 2

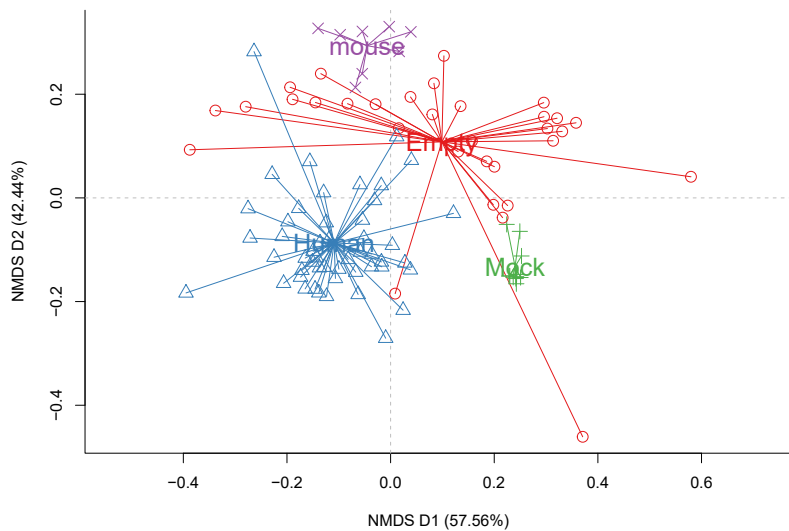**B**

Triplot - 2D NMDS - Dim 1 &amp; 2

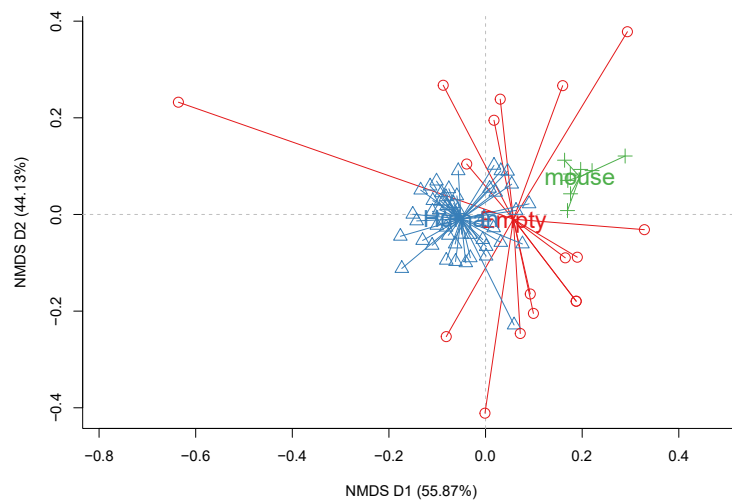**C**

Triplot - 2D NMDS - Dim 1 &amp; 2

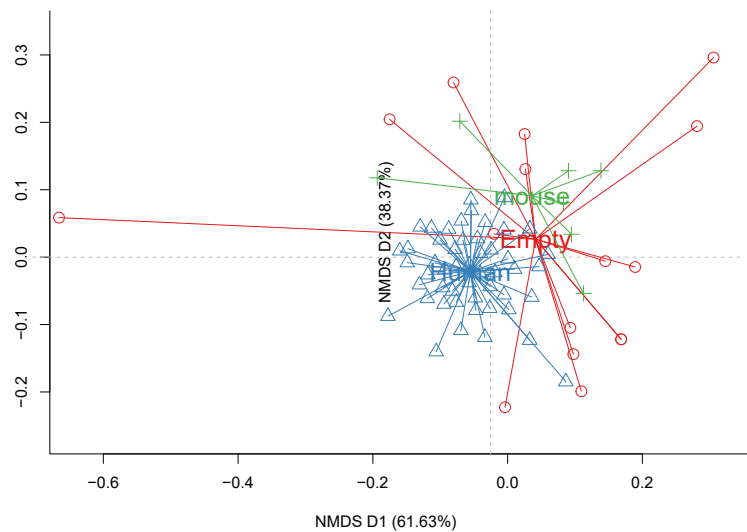

Supplement: Supplementary file 5 — Additional file 4: Suppl. Figure 4. Non-metric multidimensional scaling (NMDS). NMDS ordination of study groups (A) before any, (B) after automatic contamination and off-target removal, and (C) after removal of additional off-target zOTUs (N=75, Suppl. Fig. 7). Distance between study groups diminishes after computational removal approaches. Note that additional off-targets and contaminants later described in the text and Suppl. Fig. 7 are still included in B. Symbols represent samples, and distances between symbols represent similarities, i.e. closer symbols are more similar than distant symbols. Human, all human samples; Mouse, all murine samples; Empty, all negative controls; Mock, mock community samples. [file 40168_2021_1012_MOESM5_ESM.pdf]

## DNA\_Buffer

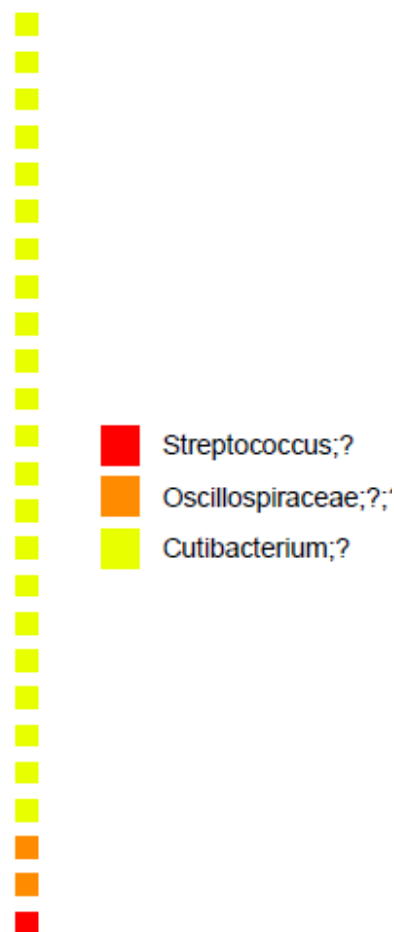

## sterileWater

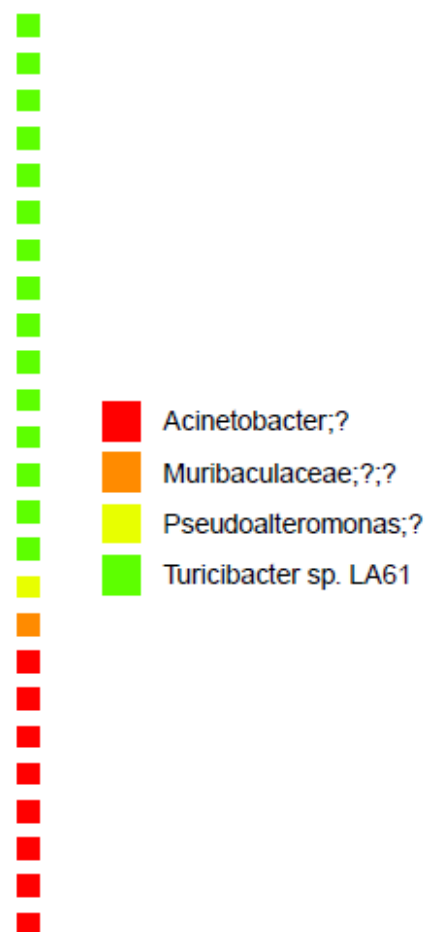

## KitomeUKB

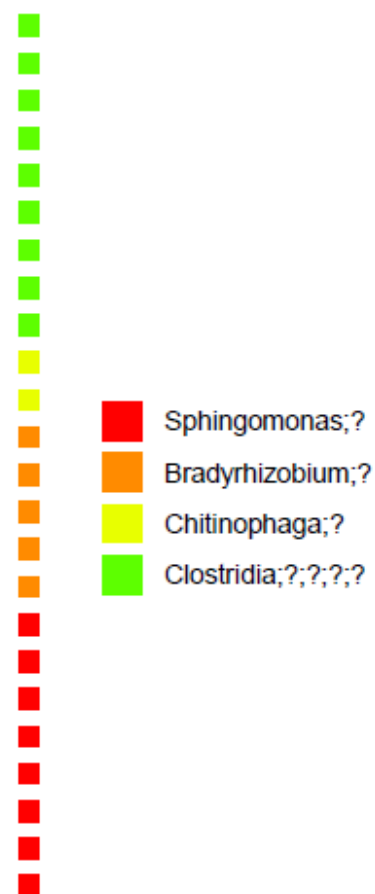

## KitomeQIB

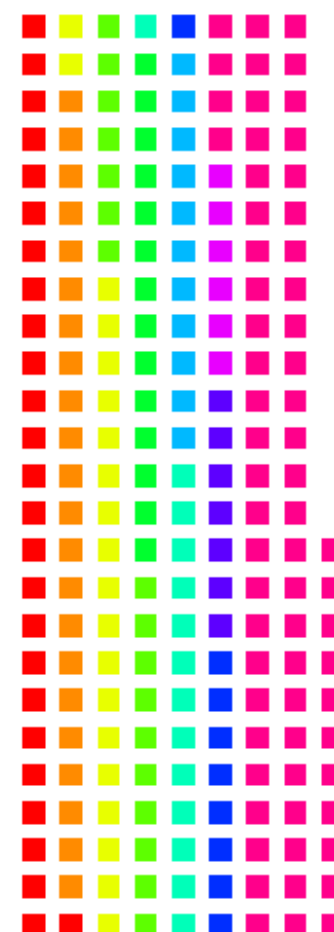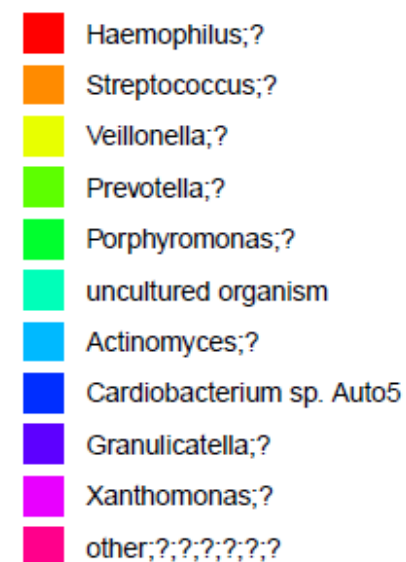

Supplement: Supplementary file 6 — Additional file 5: Suppl. Figure 5. Negative controls. Taxonomic composition (genus level) of negative controls normalized by 16S rRNA gene copy number; further analysis showed 99% of all zOTUs in negative control samples were attributable to contaminant bacteria, while <1% represented human DNA (only present in KitomeUKB/DNA extraction blank control, possibly representing cross-talk or human contaminants). Most taxa were introduced during the sequencing process, thus, library preparation and PCR amplification (KitomeQIB/no template control). Each square represents one 16S rRNA gene copy/μl. KitomeUKB, negative control of reagents used in the UKB, Bonn, Germany. KitomeQIB, negative control of reagents used in the QIB, Norwich, UK. [file 40168_2021_1012_MOESM6_ESM.pdf]

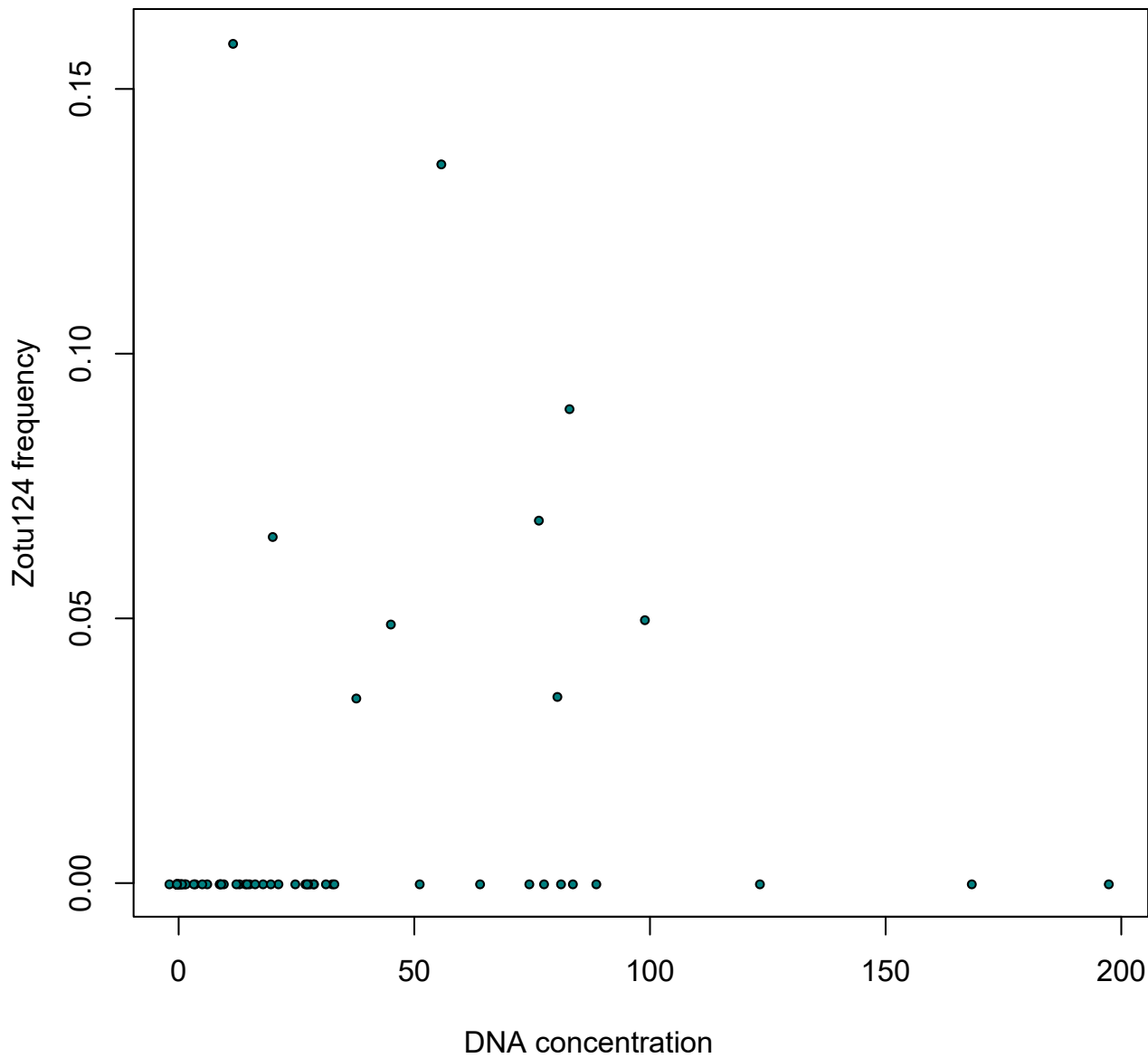

Supplement: Supplementary file 7 — Additional file 6: Suppl. Figure 6. Frequency of zOTU124 in relation to the bacterial biomass assessed with 16S rRNA gene qPCR strengthens interpretation of contamination origin. [file 40168_2021_1012_MOESM7_ESM.pdf]

## Additional Zotus Classifier Phylum level

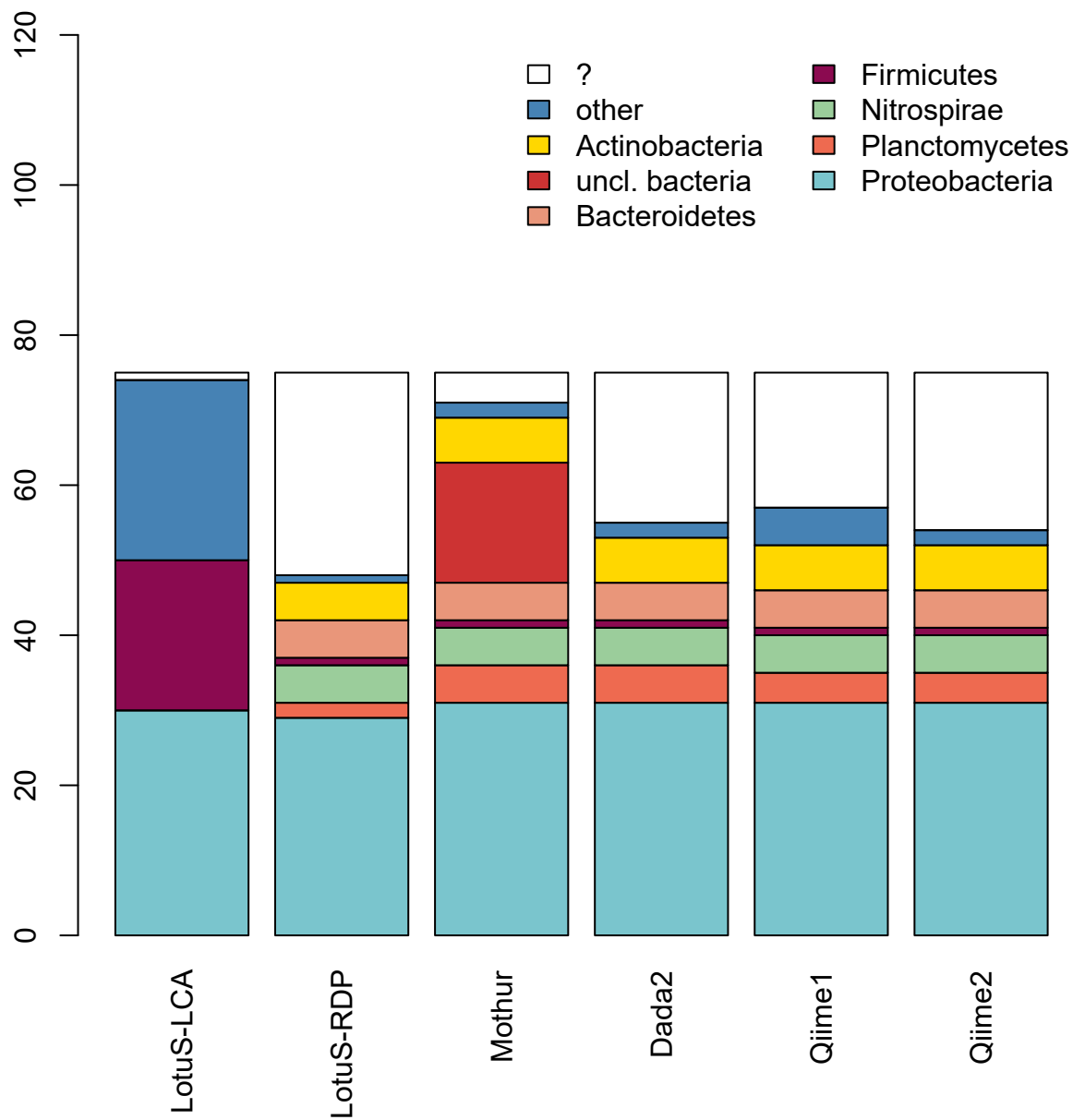

Supplement: Supplementary file 8 — Additional file 7: Suppl. Figure 7. Classification of remaining and manually re-analysed zOTUs. The 75 zOTUs that remained in the analysis after automatic off-target removal and automatic contamination removal were discovered to be off-target amplicons through manual blast searches. These 75 zOTUs were again misclassified as bacterial phyla by most amplicon pipelines; note that only zOTUs are shown, which had already been processed using the LotuS-LCA approach, thus the majority of unclassified zOTUs had already been excluded. H, human; M, mouse; Qiime1-s, Qiime1 sortmerna, Qiime1-u, Qiime1 uclust; ?, unclassified. [file 40168_2021_1012_MOESM8_ESM.pdf]
