# Supplementary material for: Much ado about nothing? Off-target amplification can lead to false-positive bacterial brain microbiome detection in healthy and Parkinson’s disease individuals
Source: Microbiome. 2021 Mar 26;9:75. doi: 10.1186/s40168-021-01012-1 (PMC8004470; doi:10.1186/s40168-021-01012-1)
Supplement: Supplementary file 9 — Additional file 8: Suppl. Table 1. Published contaminants from ‘blank controls’ (according to Salter et al .[37] and Eisenhofer et al., 2019 [16] [file 40168_2021_1012_MOESM9_ESM.docx]

**Suppl. Table 1, published contaminants introduced by ‘blank controls’**

| **Genus** | Afipia, Aquabacterium, Asticcacaulis, Aurantimonas, Beijerinckia, Bosea, Bradyrhizobium, Brevundimonas, Caulobacter,  Craurococcus, Devosia, Hoeflea, Mesorhizobium, Methylobacterium, Novosphingobium, Ochrobactrum, Paracoccus,  Pedomicrobium, Phyllobacterium, Rhizobium, Roseomonas, Sphingobium, Sphingomonas, Sphingopyxis  Acidovorax, Azoarcus, Azospira, Burkholderia, Comamonas, Cupriavidus, Curvibacter, Delftia, Duganella, Herbaspirillum, Janthinobacterium, Kingella, Leptothrix, Limnobacter, Massilia, Methylophilus, Methyloversatilis, Oxalobacter, Pelomonas, Polaromonas, Ralstonia, Schlegelella, Sulfuritalea, Undibacterium, Variovorax  Acinetobacter, Enhydrobacter, Enterobacter, Escherichia, Nevskia, Pseudomonas, Pseudoxanthomonas, Psychrobacter, Stenotrophomonas, Xanthomonas  Aeromicrobium, Arthrobacter, Beutenbergia, Brevibacterium, Corynebacterium, Curtobacterium, Dietzia,  Geodermatophilus, Janibacter, Kocuria, Microbacterium, Micrococcus, Microlunatus, Patulibacter, Propionibacterium,  Rhodococcus, Tsukamurella  Abiotrophia, Bacillus, Brevibacillus, Brochothrix, Facklamia, Paenibacillus, Streptococcus, Chryseobacterium,  Dyadobacter, Flavobacterium, Hydrotalea, Niastella, Olivibacter, Pedobacter, Wautersiella, Thermus Deinococcus,  Actinomyces, Rothia  Atopobium, Sediminibacterium, Porphyromonas, Prevotella, Capnocytophaga, TM7, Geobacillus, Staphylococcus,  Granulicatella, Enterococcus, Lactobacillus, Clostridium, Coprococcus  Anaerococcus, Dialister, Megasphaera, Veillonella, Fusobacterium, Leptotrichia, Achromobacter, Kingella, Neisseria,  Haemophilus |
| --- | --- |

Published contaminants from ‘blank controls’ (according to Salter et al.[38] and Eisenhofer et al., 2019 [17]
