# Supplementary material for: Much ado about nothing? Off-target amplification can lead to false-positive bacterial brain microbiome detection in healthy and Parkinson’s disease individuals
Source: Microbiome. 2021 Mar 26;9:75. doi: 10.1186/s40168-021-01012-1 (PMC8004470; doi:10.1186/s40168-021-01012-1)
Supplement: Supplementary file 11 — Additional file 10: Suppl. Table 3. Overview of different classes of contaminants identified and corrected for bioinformatically. [file 40168_2021_1012_MOESM11_ESM.docx]

**Suppl. Table 3, Overview of different classes of zOTUs identified and corrected for bio-informatically**

|  | **True zOTU** | **False zOTU** |
| --- | --- | --- |
| **True biological**  (Present in original sample) | Putative bacteria in sample  (see Fig. 4a, b) | Off-targets  (see Fig. 1 and Suppl. Fig.2 and Suppl. Fig. 7) |
| **False biological** | Contaminant bacteria, exogenous DNA (environment), Cross-talk  (see Fig. 2 and Suppl. Fig. 2b and Suppl. Fig. 3) | Sequencing-noise, Chimeric |
