# Supplementary material for: Much ado about nothing? Off-target amplification can lead to false-positive bacterial brain microbiome detection in healthy and Parkinson’s disease individuals
Source: Microbiome. 2021 Mar 26;9:75. doi: 10.1186/s40168-021-01012-1 (PMC8004470; doi:10.1186/s40168-021-01012-1)
Supplement: Supplementary file 12 — Additional file 11: Suppl. Table 4. qPCR-derived 16S rRNA gene copies in brain tissue and control samples. PD, Parkinson’s disease, HC, healthy control; SC, sterile control; GRF, germ free; SPF, specific pathogen free; KitUKB, empty tube; BF, Tris-HCl buffer; KitQIB, PCR reagents; SW, sterile/autoclaved water; NA, not applicable; Data is presented as the median. P-values are given for the final 16S rRNA gene copy numbers; **P=3.15x10^-6^ vs. controls; P=0.0018 vs. mouse; *P=0.0014 vs controls. [file 40168_2021_1012_MOESM12_ESM.docx]

**Suppl. Table 4, 16S rRNA qPCR gene copies in brain tissue and control samples**

| **Group** | **Copies/mg tissue**  **Blank corrected/off target corrected** | **Group** | **Copies/mg tissue**  **Blank corrected/ off target**  **corrected** |
| --- | --- | --- | --- |
| Human | 122.56/27.72 ** | PD | 132.92/32.25 |
|  |  | HC | 101.03/16.01 |
|  |  | SC | 195.50/38.07 |
| Mouse | 13.72/1.38* | GRF | 5.41/0.86 |
|  |  | SPF | 14.71/1.91 |
| Controls | **Copies/µl sample input**  **Blank corrected*/ off target* corrected** |  | **Copies/µl sample input**  **Blank corrected/ off target corrected** |
|  | 152.61/0.00 | KitUKB | 0.00/0.00 |
|  |  | BF | 11.54/0.00 |
|  |  | SW | 17.40/0.00 |
|  |  | KitQIB | 169.9/NA |

PD, Parkinson’s disease, HC, healthy control; SC, sterile control; GRF, germ free; SPF, specific pathogen free; KitUKB, empty tube; BF, Tris-HCl buffer; KitQIB, PCR reagents; SW, sterile/autoclaved water; NA, not applicable; Data is presented as the median. P-values are given for the final 16S rRNA gene copy numbers; **P=3.15x10-6 vs. controls; P=0.0018 vs. mouse; *P=0.0014 vs controls.
